# *DUX4* is a multifunctional factor priming human embryonic genome activation

**DOI:** 10.1016/j.isci.2022.104137

**Published:** 2022-03-22

**Authors:** Sanna Vuoristo, Shruti Bhagat, Christel Hydén-Granskog, Masahito Yoshihara, Lisa Gawriyski, Eeva-Mari Jouhilahti, Vipin Ranga, Mahlet Tamirat, Mikko Huhtala, Ida Kirjanov, Sonja Nykänen, Kaarel Krjutškov, Anastassius Damdimopoulos, Jere Weltner, Kosuke Hashimoto, Gaëlle Recher, Sini Ezer, Priit Paluoja, Pauliina Paloviita, Yujiro Takegami, Ai Kanemaru, Karolina Lundin, Tomi T. Airenne, Timo Otonkoski, Juha S. Tapanainen, Hideya Kawaji, Yasuhiro Murakawa, Thomas R. Bürglin, Markku Varjosalo, Mark S. Johnson, Timo Tuuri, Shintaro Katayama, Juha Kere

**Affiliations:** 1 Department of Biosciences and Nutrition, Karolinska Institutet, 17177 Huddinge, Sweden; 2 Department of Obstetrics and Gynecology, 00014, University of Helsinki and Helsinki University Hospital, 00290 Helsinki, Finland; 3 RIKEN Center for Integrative Medical Sciences, Yokohama 230-0045, Japan; 4 Instutute for the Advanced Study of Human Biology, Kyoto University, Kyoto 606-8501, Japan; 5 Reproductive Medicine Unit, Helsinki University Hospital, 00290 Helsinki, Finland; 6 Institute of Biotechnology, University of Helsinki, 00790 Helsinki, Finland; 7 Stem Cells and Metabolism Research Program, University of Helsinki, 00014 Helsinki, Finland; 8 Structural Bioinformatics Laboratory, Biochemistry, Faculty of Science and Engineering, Åbo Akademi University, 20520 Turku, Finland; 9 Competence Centre for Health Technologies, 51010 Tartu, Estonia; 10 University of Tartu, Department of Obstetrics and Gynecology, Institute of Clinical Medicine, 50406 Tartu, Estonia; 11 Karolinska Institutet, Bioinformatics and Expression Analysis Core Facility, 17177 Huddinge, Sweden; 12 Laboratoire Photonique Numérique et Nanosciences, CNRS, Institut d’Optique Graduate School, University of Bordeaux, UMR 5298, 33400 Bordeaux, France; 13 Folkhälsan Research Center, 00290 Helsinki, Finland; 14 Institute of Clinical Medicine, University of Tartu, 50090 Tartu, Estonia; 15 University of Helsinki, Doctoral Program in Population Health, 00014 Helsinki, Finland; 16 K.K. DNAFORM, Yokohama 230-0051, Japan; 17 Children’s Hospital, Helsinki University Central Hospital, 00290; 18 Oulu University Hospital, 90220 Oulu, Finland; 19 RIKEN Preventive Medicine and Diagnosis Innovation Program, Wako 351-0198, Japan; 20 Tokyo Metropolitan Institute of Medical Science, Tokyo 156-8506, Japan; 21 IFOM, The FIRC Institute of Molecular Oncology, 20139 Milan, Italy; 22 Department of Medical Systems Genomics, Graduate School of Medicine, Kyoto University, Kyoto 606-8501, Japan; 23 Department of Biomedicine, University of Basel, 4031 Basel, Switzerland

**Keywords:** developmental biology, biology of human development, molecular biology

## Abstract

Double homeobox 4 (*DUX4*) is expressed at the early pre-implantation stage in human embryos. Here we show that induced human *DUX4* expression substantially alters the chromatin accessibility of non-coding DNA and activates thousands of newly identified transcribed enhancer-like regions, preferentially located within ERVL-MaLR repeat elements. CRISPR activation of transcribed enhancers by C-terminal DUX4 motifs results in the increased expression of target embryonic genome activation (EGA) genes *ZSCAN4* and *KHDC1P1*. We show that *DUX4* is markedly enriched in human zygotes, followed by intense nuclear DUX4 localization preceding and coinciding with minor EGA. *DUX4* knockdown in human zygotes led to changes in the EGA transcriptome but did not terminate the embryos. We also show that the DUX4 protein interacts with the Mediator complex via the C-terminal KIX binding motif. Our findings contribute to the understanding of *DUX4* as a regulator of the non-coding genome.

## Highlights

• DUX4 induces the expression of thousands of accessible, transcribed putative enhancers
• *DUX4* RNA is enriched in human zygotes; the protein is enriched in the nucleus before EGA
• *DUX4* knockdown in human zygotes leads to minor embryonic transcriptome changes
• DUX4 protein interacts with the Mediator complex via the KIX binding motif

## Graphical abstract

**Figure:**
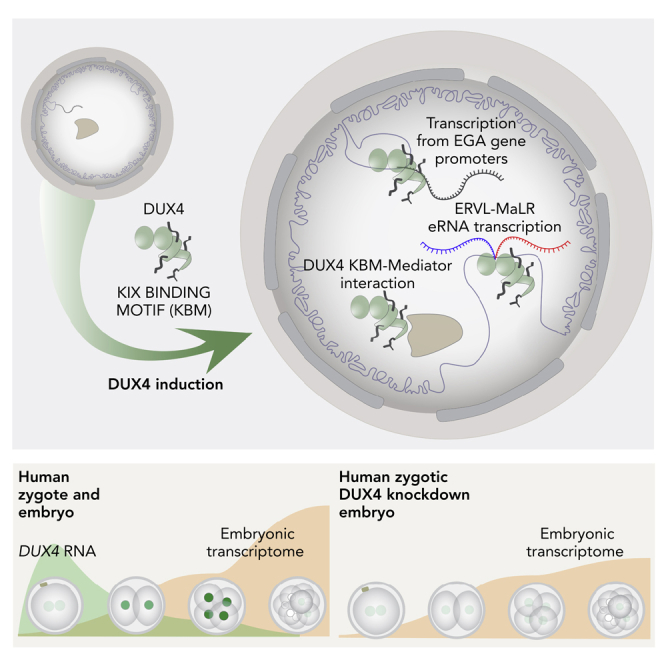

## Introduction

Mammalian pre-implantation development commences with conversion of the differentiated gametes into a totipotent zygote. Successful reprogramming of the zygote involves prominent chromatin remodeling and changes in epigenetic landscapes (Conti and Franciosi, 2018; Jukam et al., 2017; Li et al., 2018). Chromatin of the human mature oocyte is essentially inaccessible and transcriptionally silent, whereas progressive increase in chromatin accessibility commences soon after fertilization (Li et al., 2018; Liu et al., 2019; Wu et al., 2018). Embryonic genome activation (EGA) occurs in minor and major transcription waves. Minor EGA involves pervasive but low-level transcription that is necessary for pre-implantation development in mouse (Abe et al., 2015, 2018; Aoki et al., 1997; Zeng and Schultz, 2005). The minor and major EGA waves take place in humans at 4-cell and 8-cell stages, respectively (Braude et al., 1988; Dobson et al., 2004; Tesarik et al., 1987; Tohonen et al., 2015). The gene expression profile at the time of maternal-to-zygotic transition differs from that of later embryonic stages, involving transcription from non-coding genomic loci that are predominantly expressed in cleavage stage embryos (Kigami et al., 2003; Peaston et al., 2004; Tohonen et al., 2015).

The conserved *DUX*-family transcription factors are expressed in several mammalian cleavage stage embryos, including mouse and primate (Whiddon et al., 2017). Recent findings have suggested that *DUX* may act as a pioneer transcription factor in mammals (De Iaco et al., 2017; Hendrickson et al., 2017) similar to Zelda in *Drosophila melanogaster* (Liang et al., 2008; McDaniel et al., 2019). *Dux* knockout mice can survive until adulthood (Chen and Zhang, 2019) but litter sizes from these animals are significantly reduced, indicating cumulative defects over generations (De Iaco et al., 2020). *Ex vivo* culture of *Dux* knockout mouse embryos revealed delayed development beyond the genome activation stage with only 65% of the knockout embryos reaching the blastocyst stage at E4.5 (De Iaco et al., 2020). *DUX4* is expressed in early human embryos (De Iaco et al., 2017; Hendrickson et al., 2017) and the DUX4 binding motif is enriched at the promoter regions of the human EGA genes, such as *LEUTX* (at the 4-cell stage), and *ARGFX*, *DPRX*, and *TPRXs* (at the 8-cell stage) (Hendrickson et al., 2017; Tohonen et al., 2015), suggesting a key role for *DUX4* in human genome activation (De Iaco et al., 2017; Geng et al., 2012; Hendrickson et al., 2017).

In addition to protein coding transcripts, *DUX*-family transcription factors activate transcription from non-coding repeat elements (Geng et al., 2012; Whiddon et al., 2017; Young et al., 2013). Mouse Dux and human DUX4 transcription factors diverge on their homeodomain structure, correlating with their species specificity on retrotransposon activation (Whiddon et al., 2017). *DUX4* activates transcription from ACRO1 and HSATII satellite repeats, as well as from the long terminal repeat (LTR)-containing elements (De Iaco et al., 2017; Hendrickson et al., 2017; Liu et al., 2019; Whiddon et al., 2017). Accumulating data indicate that repeat loci have been evolutionarily co-opted as regulatory elements for gene expression (Feschotte, 2008; Gerdes et al., 2016; Pontis et al., 2019; Thompson et al., 2016) and that particular repeat families have contributed to the evolution of gene regulatory networks; for example, in placentation (Chuong, 2013) and pregnancy (Lynch et al., 2011). Although transcriptional activation of LTR elements in human embryos (Goke et al., 2015; Grow et al., 2015; Hashimoto et al., 2021) and their invocation as alternative promoters have been established (Franke et al., 2017; Whiddon et al., 2017), broader implications of the *DUX4*-activated repeat elements in the context of human embryo development are largely unexplored.

Enhancers are short DNA regions that are typically characterized by depletion of nucleosomes, overlap with DNAse I hypersensitivity sites (DHS), and being flanked by specific histone modifications (Murakawa et al., 2016). Active enhancers generate RNAs in a bidirectional manner and they are usually positive for H3K27ac and H3K4me1 (Andersson et al., 2014; Arner et al., 2015; Henriques et al., 2018; Hirabayashi et al., 2019; Hon et al., 2017). Transcribed enhancers have a higher tendency of being functionally validated in reporter experiments when compared to non-transcribed enhancers identified only by using histone modifications or DHSs (Andersson et al., 2014). Indeed, functional enhancer units are precisely defined by active transcription start sites (Tippens et al., 2020). Recent analyses show that distal accessible chromatin regions in human early embryos overlap with oocyte hypomethylated regions, transposable elements, and putative *cis*-regulatory elements (Wu et al., 2018). Here, we elucidated the dynamics and involvement of *DUX4* during the human EGA process and shed light on how newly identified *DUX4*-activated *cis*-regulatory elements regulate human EGA transcripts.

## Results

### DUX4 activates thousands of newly identified bidirectionally transcribed enhancer-like regions that are enriched for ERVL-MaLR repeats

**Figure 1:**
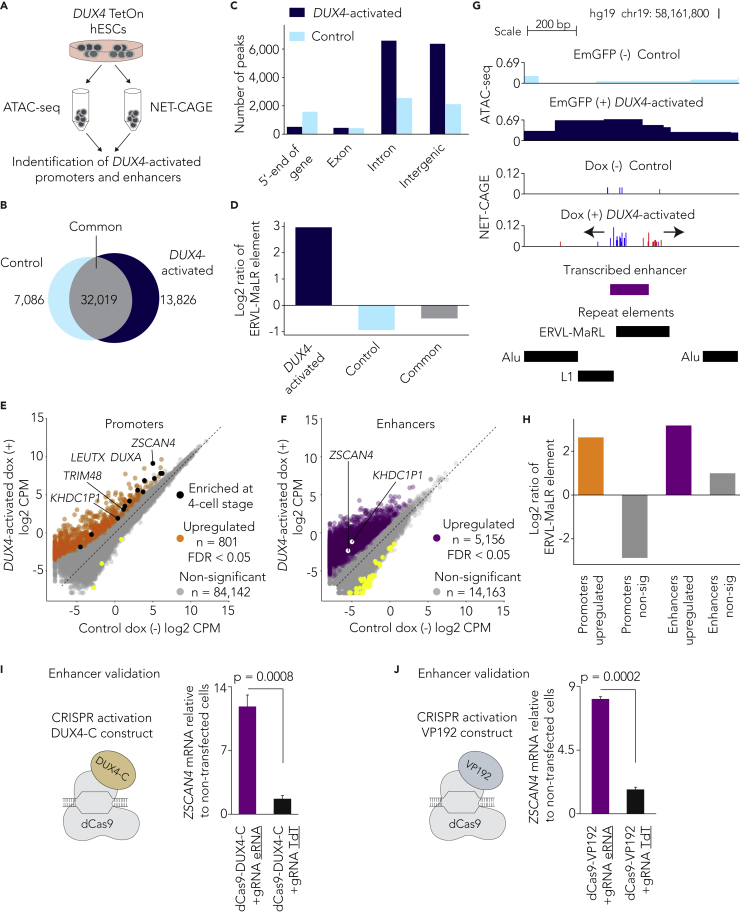
DUX4 activates thousands of newly identified bidirectionally transcribed enhancer-like regions that are enriched for ERVL-MaLR repeats (A) Schematic of the experimental outline. hESCs carrying an inducible *DUX4*-TetOn construct were doxycycline (dox) induced for 4 h. ATAC-seq and NET-CAGE were performed to identify accessible and transcribed *cis*-regulatory elements, respectively. (B) Venn diagram showing the number of ATAC-seq peaks in control and *DUX4*-activated hESC. (C) Bar plot showing the distribution of ATAC–seq peaks in control and *DUX4*-activated cells across the genome. (D) Bar plot showing the log2 ratio of ATAC–seq peaks overlapping ERVL-MaLR regions over randomly selected background regions (See STAR Methods). (E and F) Global differential expression analysis of *DUX4*-expressing (dox +) and control (dox -) hESCs for promoters (E) and putative enhancers (F). Log2 mean (counts per million, CPM) of four *DUX4*-expressing (dox +) and four control (dox -) replicates has been shown. Orange and purple dots indicate significantly upregulated (FDR < 0.05) promoters (E) and putative enhancers (F), respectively. Black dots indicate promoters for known 4-cell stage embryo genome activation genes. White dots indicated enhancers validated using the CRISPR activation assay. Yellow dots indicate significantly downregulated (FDR < 0.05) promoters (E) and putative enhancers (F), respectively. Grey dots indicate non-significantly differentially expressed promoters (E) and putative enhancers (F). (G) Genome browser view showing the putative enhancer-like region for *ZSCAN4*. The promoter for *ZSCAN4* is 20.5 kb downstream of the putative enhancer. ATAC-seq signal indicates the accessibility of chromatin and NET-CAGE signal shows bidirectional transcription start sites of enhancer RNAs in dox (+) hESCs. NET-CAGE reads in red, plus strand; NET-CAGE reads in blue, minus strand. The putative enhancer also overlaps ERVL-MaLR repeat element. See also Figure S5. (H) Bar plot showing the log2 ratio of promoters and putative enhancers overlapping ERVL-MaLR regions over randomly selected background regions. (I and J) Schematic of CRISPR dCas9 activator constructs fused with DUX4 C-terminal end (I) or VP192 (J) used in combination with guide RNA pools to activate putative enhancers. Graphs show *ZSCAN4* expression level relative to non-transfected cells (n = 6 from independent cell cultures (I); n = 3 from independent cell cultures (J)). Guide RNA construct for TdT were used as negative control. Data are shown as mean ± SD and p-values were calculated using two-tailed Student’s *t*-test. See also Figures S1–S5.

To extend previous analyses on chromatin accessibility and repeat elements in human embryos (Goke et al., 2015; Hendrickson et al., 2017; Li et al., 2018; Liu et al., 2019; Whiddon et al., 2017; Wu et al., 2018), we first identified loci that are associated with *DUX4* expression. To this end, we performed the assay for transposase-accessible chromatin with high-throughput sequencing (ATAC-seq) (Buenrostro et al., 2015) using doxycycline-inducible *DUX4*-TetOn human embryonic stem cells (hESC) (Figures 1A and S1A–S1D). Our analyses revealed substantial changes in the chromatin landscape of *DUX4*-activated hESCs after only a 4-h doxycycline treatment. We detected 13,826 peaks that were accessible only in *DUX4*-activated cells while 7,086 peaks were accessible only in control cells (Figure 1B). The majority of the *DUX4*-activated peaks overlapped intronic and intergenic regions indicating that the non-coding genome had become accessible (Figures 1C and S2A). Gene ontology (GO) analysis for biological processes suggested that *DUX4*-activated peaks are associated with developmental processes including myotube differentiation (Figure S2B). Integration of the ATAC-seq peaks with repeat elements showed ∼3-fold enrichment of ERVL-MaLR repeats (belonging to the LTR family) in *DUX4*-activated peaks but depletion in control peaks (Figures 1D and S2C). The notable enrichment of non-coding ERVL-MaLR elements prompted us to study bi-directionally transcribed enhancer-like regions using native elongating transcript – cap analysis of gene expression (NET-CAGE) with high-throughput sequencing (Figure S3A) (Hirabayashi et al., 2019) in *DUX4*-TetOn hESCs (Figure 1A). Altogether, we identified ∼2M transcription start site (TSS) clusters of which ∼ 200,000 mapped to 5’ -ends of genes (also referred to as promoters) and ∼1.3M mapped to intronic and intergenic regions (Figure S3B). After excluding lowly expressed TSS clusters, we identified 84,946 promoters and 19,358 bi-directionally transcribed enhancer-like regions (Table S1) that correlated well between biological replicates (Figure S3C). Remarkably, only 10.4% of *DUX4*-activated putative enhancers-like regions were also observed in other cell-types and tissues indicating the cell-type-specific nature of transcribed enhancers (Andersson et al., 2014; Arner et al., 2015; Hirabayashi et al., 2019). Comparison of control and *DUX4*-activated hESCs showed significant upregulation (FDR < 0.05) of 801 promoters (Table S1), which included known EGA genes such as *ZSCAN4*, *DUXA*, and *LEUTX* as well as recently annotated genes such as *KHDC1P1* (Tohonen et al., 2015) (Figure 1E). We also observed the significant upregulation (FDR < 0.05) of 5,156 putative enhancer-like regions (Figures 1F, 1G and Table S1) of which ∼50% also overlapped *DUX4*-activated ATAC-seq peaks (Figure S3D). Similar to *DUX4*-activated ATAC-seq peaks, significantly upregulated promoters and enhancer-like regions were also enriched for ERVL-MaLR repeat elements (Figures 1G, 1H, S3E and S3F). Consistent with previous findings (Geng et al., 2012; Liu et al., 2019; Whiddon et al., 2017; Young et al., 2013), our result emphasizes ERVL-MaLRs as repeat elements that potentially contribute regulatory accessible regions and transcripts for the human EGA genes.

### Putative *DUX4* target genes cloned from human 4-cell stage embryos

Purification of millions of cells for NET-CAGE (Hirabayashi et al., 2019) using fluorescence activated cell sorting (FACS) was not feasible. Therefore, we separated the *DUX4* expressing (EmGFP+) and control (EmGFP-) hESCs by FACS and performed bulk RNA-seq using the modified single-cell tagged reverse transcription (STRT) method (Krjutskov et al., 2016b). Comparison of mRNA levels in *DUX4*-activated and in control cells confirmed the significant upregulation of known EGA genes such as *ZSCAN4* as well as the three recently annotated genes –*KHDC1P1*, *RETT FINGER PROTEIN*, and *RING FINGER PROTEIN* (Figure S4A and Table S2) (Tohonen et al., 2015) – in the DUX4-positive cells. These annotated genes are expressed in cleavage stage human embryos (Tohonen et al., 2015). We cloned the predicted cDNAs from human 4-cell stage embryos (Figure S4B–S4D), confirming the presence of these transcripts in cleavage stage embryos.

### Functional validation of *DUX4*-activated enhancer-like regions

The CAGE-based cap-trapping method (Murata et al., 2014) allowed us to pinpoint the TSS of the *ZSCAN4* (Figure S5A), and *KHDC1P1* (Figure S5B) promoters at nucleotide resolution. Annotation of the bidirectionally transcribed enhancer-like regions that were significantly upregulated after *DUX4* expression revealed a potential enhancer for *ZSCAN4* (Figures 1F and 1G). The putative *ZSCAN4* enhancer (Figure 1G) is located around 20 kb from the *ZSCAN4* promoter (Figure S5A). The putative *ZSCAN4* enhancer is also accessible in *DUX4*-activated cells but not in control cells, and it overlaps an ERVL-MaLR repeat element (Figure 1G). To test the functionality of enhancer-like regions using CRISPR activation, we first generated a dCas9–DUX4 C-terminal fusion protein, which contains the DUX4 C-terminal 9aaTAD and KIX-binding motif (KBM) (but not the DUX4 N-terminal DNA-binding homeodomains), fused with endonuclease deficient dCas9 (hereafter dCas9–*DUX4*-C; Figures 1I and S5C). We used either dCas9-*DUX4*-C or the conventional VP16 *trans* activator domains containing dCas9-VP192 (Weltner et al., 2018) construct (Figures 1I and 1J) in combination with guide RNA (gRNA) pools to target the putative enhancer-like regions in HEK293 cells. We designed altogether five gRNAs (key resources table) for the *ZSCAN4* enhancer region to experimentally test the capacity of this enhancer to activate expression of the putative target gene, *ZSCAN4.* Activation of the *ZSCAN4* enhancer region, using both the dCas9-*DUX4*-C and the dCas9-VP192 construct with a pool of gRNAs, led to significant upregulation of the *ZSCAN4* expression level, in comparison with the respective controls, dCas9-*DUX4*-C with TdT guide RNA construct (p = 0.0008, two-tailed Student’s *t*-test Figure 1I) or dCas9-VP192 with TdT guide RNA construct (p = 0.0002, two-tailed Student’s *t*-test Figure 1J). Similarly, we also tested the *KHDC1P1* enhancer region (Figures 1F and S5D). Activation of the *KHDC1P1* enhancer region (using both constructs) led to significant upregulation of the *KHDC1P1* expression level (Figures S5E and S5F). These findings reveal the functionality of specific *DUX4*-activated transcribed enhancers.

### DUX4 expression dynamics and localization of the DUX4 protein in human zygotes and early embryos

**Figure 2:**
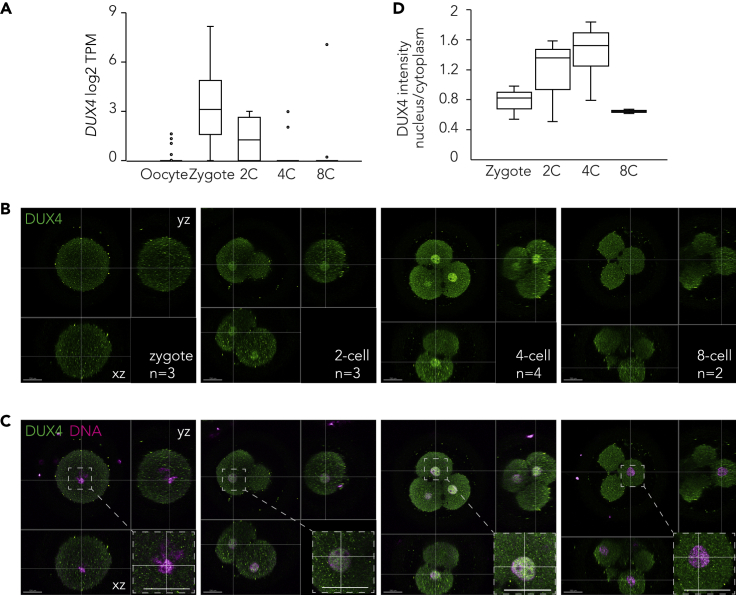
*DUX4* expression dynamics and localization of the DUX4 protein in human zygotes and early embryos (A) Bar plot showing the log2 transcripts per million (TPM) of DUX4 mRNA reads in human MII oocytes (n = 20), zygotes (n = 59), 2-cell (n = 4), 4-cell (n = 15), and 8-cell (n = 14) embryos. Source data Tohonen et al., (2015). A pseudo count of 1 was added. (B and C) Human diploid zygotes (n = 3), 2-cell (n = 3), 4-cell (n = 4), and 8-cell (n = 2) embryos were immunostained with monoclonal DUX4 antibody (green) (B), and nuclei were counterstained with DAPI (magenta) (C). Orthogonal views along the depicted lines are shown (B, C). On the lower right corner (C) a magnification of the indicated region is shown. Scale bars 130 μm. (D) A box plot showing quantification of the DUX4 staining intensity in the nucleus in 3D normalized to the intensity in the cytoplasm. The samples are as described in (B and C). In each box the median is indicated, the edges are the 25^th^ and 75^th^percentiles, the whiskers extend to the data points not considered outliers. See also Figure S6 and Videos S1, S2, S3, and S4.

To study the expression of *DUX4* in human embryos, we utilized our published STRT sequencing data (Tohonen et al., 2015) that identified 5’ transcript far ends (TFEs) in human metaphase II (MII) oocytes, zygotes, 2-cell, 4-cell and 8-cell stage embryos, and observed enrichment of *DUX4* mRNA in zygotes (Figure 2A). In previous studies, *DUX4* mRNA has been observed in either 4-cell stage embryos (Hendrickson et al., 2017) or more broadly throughout the cleavage stages (Dang et al., 2016; Xue et al., 2013; Yan et al., 2013) (Figure S6A). Timely differences in observed *DUX4* enrichment could be due to different sequencing methods that rely on quantification of the 5’ -end (Tohonen et al., 2015) or 3’ -end (Hendrickson et al., 2017; Yan et al., 2013) of mRNAs. Zygotic enrichment of the *DUX4* mRNA orthologs is evolutionarily conserved in mouse (Figure S6B) and non-human primates (Figure S6C), suggesting that *DUX4* is likely to be important at the time of genome activation in mammals. Given that *DUX4* can activate EGA genes in humans (De Iaco et al., 2017; Hendrickson et al., 2017; Liu et al., 2019), and that *DUX4* activates ERVL-MaLR-enriched nascent enhancer RNAs, we next characterized DUX4 protein localization in early human embryos. We observed an overall increase in DUX4 antibody staining from zygote to 2-cell stage, and further to 4-cell stage and rapid clearance at the 8-cell stage (Figures 2B and 2C). DUX4 staining was observed both in the cytoplasm and nucleus and we therefore quantified the nuclear DUX4 staining intensities from the three-dimensional confocal stacks. Quantifications revealed variable but increasing nuclear signals from zygotes up to 4-cell stage embryos, while only a weak signal was detected in the nuclei of 8-cell stage embryos (Figure 2C insets and 2D). Supplemental 3D movies of unprocessed immunofluorescence stainings show DUX4 localization in the nuclei over the developmental trajectory from zygotes to 8-cell stage (Videos S1, S2, S3, and S4). Our analyses show that *DUX4* transcripts become abundant after fertilization and rapidly reduce in 2-cell and 4-cell stage embryos. Nuclear localization of the DUX4 protein peaks during the first two days of human embryo development coincided with the onset of EGA.

Video S1. Representative movie of a human zygote immunostained for DUX4 (green) Nuclei counterstained with DAPI (magenta). Raw data without processing is shown. Related to Figure 2.

Video S2. Representative movie of a human 2-cell stage embryo immunostained for DUX4 (green) Nuclei counterstained with DAPI (magenta). Raw data without processing is shown. Related to Figure 2.

Video S3. Representative movie of a human 4-cell stage embryo immunostained for DUX4 (green) Nuclei counterstained with DAPI (magenta). Raw data without processing is shown. Related to Figure 2.

Video S4. Representative movie of a human 8-cell stage embryo immunostained for DUX4 (green) Nuclei counterstained with DAPI (magenta). Raw data without processing is shown. Related to Figure 2.

### *DUX4* knockdown in human zygotes leads to minor changes in the embryonic transcriptome

**Figure 3:**
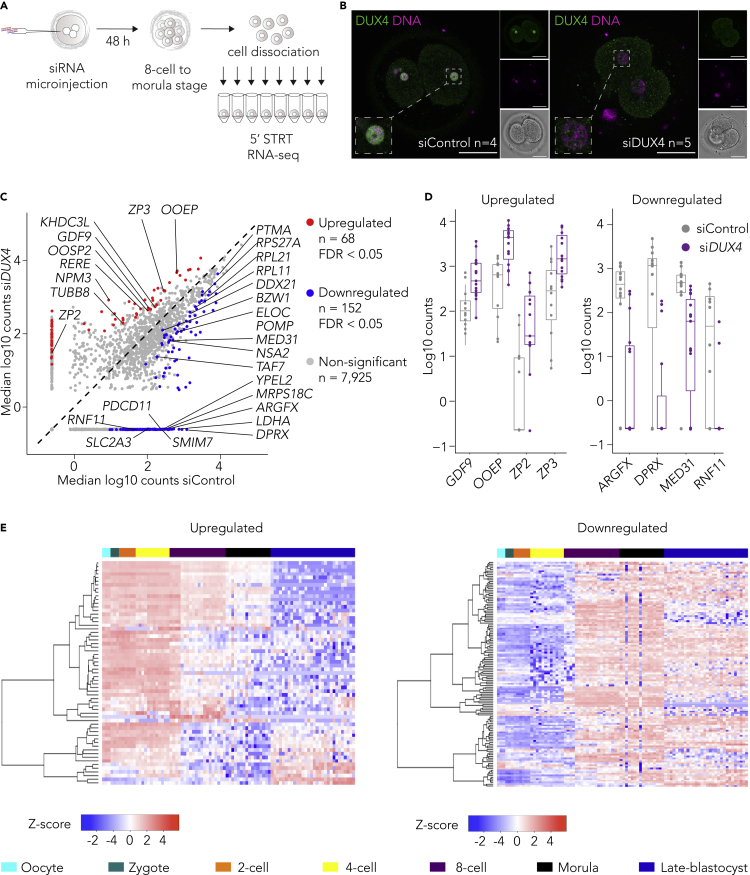
*DUX4* knockdown in human zygotes leads to minor changes in the embryonic transcriptome (A) Schematic of 5’ STRT RNA sequencing of human embryos microinjected with either control or DUX4 targeting siRNA. (B) Representative confocal images of human embryos immunostained with monoclonal DUX4 antibody (green) 24 h after microinjection with either control (n = 4 zygotes) or *DUX4* targeting (n = 5 zygotes) siRNA. Nuclei counterstained with DAPI (blue). Overlay of single DUX4 and DAPI z-planes is shown on the left together with an inset of a nucleus, and the corresponding z-planes and the bright field plane are shown on the right. Scale bars 50 μm. (C) A scatter plot showing the comparison of median log10 gene expression levels in the siControl blastomeres (n = 12 cells from two embryos) versus siDUX4 blastomeres (n = 15 cells from three embryos). Red and blue dots represent significantly upregulated and downregulated genes, respectively. Significance was calculated using Wilcoxon test, FDR < 0.05. Grey dots represent non-significantly differentially expressed genes. (D) Boxplots showing the expression levels in individual siControl and siDUX4 cells for selected oocyte-specific genes (left) and 8-cell stage genes seen during embryonic genome activation (right). All genes are statistically significant by Wilcoxon test, FDR < 0.05. In each box, the median line is indicated, the edges are the 25^th^ and 75^th^ percentiles, the whiskers extend to the data points not considered outliers. (E) Heatmaps showing *Z* score normalized RNA-seq expression levels (Yan et al., 2013) (GEO: GSE36552) for significantly upregulated (left) and downregulated (right) genes from (C). Upregulated genes (left) are mainly expressed in oocyte, zygote and 2-cell stages while downregulated genes (right) are expressed from 8-cell stage onwards. See also Figure S6.

Recent results have indicated that Dux is not necessary for mouse development (Chen and Zhang, 2019), although negative consequences of Dux knockout seem to accumulate over generations (De Iaco et al., 2020). We asked whether *DUX4* affects the transcriptional program during EGA in human embryos, and approached this question using the best available material, human triploid (3PN) zygotes. We microinjected small interfering RNAs (siRNAs) targeting *DUX4* (siDUX4) or control siRNAs (siControl) into human 3PN zygotes and monitored them until the third day of development, up to 8-cell-to-morula stage (Figure 3A). Antibody staining of the DUX4 protein was positive in the siControl embryos but faint in the siDUX4 embryos, as observed 24 h after microinjection (Figure 3B), indicating that the siRNAs targeting the *DUX4* transcripts efficiently reduced DUX4 protein levels. The siDUX4 embryos proceeded through cleavages without differences when compared with the siControl embryos. The blastomeres from the microinjected embryos were dissociated and collected for STRT RNA-seq 48 h after microinjections, on the third day of development, when the majority of the EGA transcripts are highly expressed and the maternal transcripts are lowly expressed in humans (Braude et al., 1988; De Iaco et al., 2017; Liu et al., 2019; Tesarik et al., 1987; Tohonen et al., 2015). Comparison of 8,145 genes (Table S3) across siControl (n = 12) and siDUX4 cells (n = 15) indicated that a total of 152 genes were significantly downregulated (FDR < 0.05) of which 20 were known 8-cell stage EGA genes (Tohonen et al., 2015) such as *ARGFX* and *DPRX* (Figures 3C, 3D and S6D) (Table S3). A total of 68 genes were significantly upregulated (FDR < 0.05), the majority of which are expressed in oocyte and zygotes (Yan et al., 2013), including known maternal genes such as *GDF9* (McGrath et al., 1995), *ZP2*, and *ZP3* (Canosa et al., 2017) (Figures 3C and 3D). GO analysis for biological process suggested that upregulated genes were significantly associated with regulation of reproductive process while downregulated genes were significantly associated with translation and ribonucleoprotein complex biogenesis (Figure S6E). Integration with a publicly available single-cell RNA-seq dataset (Yan et al., 2013) indicated that upregulated genes are usually expressed in oocytes, zygotes, 2-cell and 4-cell stages while downregulated genes are expressed in 8-cell, morula and late-blastocyst stages (Figure 3E). These data suggest that the knockdown of *DUX4* in human blastomeres leads to minor changes in embryonic gene expression program.

### DUX4 C-terminal KIX binding domain interacts with MED15

**Figure 4:**
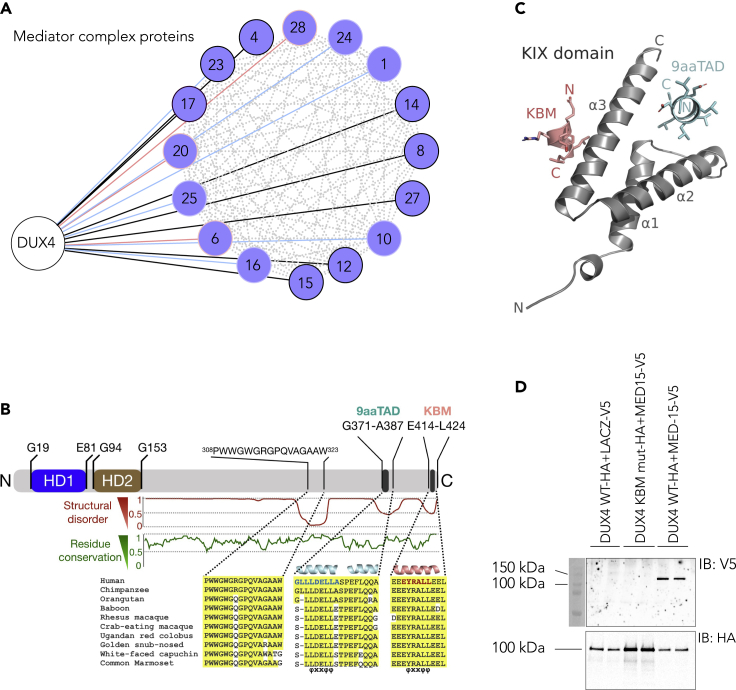
DUX4 C-terminal KIX binding domain interacts with MED15 (A) DUX4 protein-protein interactome enriched for MED protein complex is shown. BioID -interactions are shown with red lines and AP-MS -interactions are shown with blue lines. If a protein appeared in both data sets it is shown with black line and outlined in black. Known prey-prey interactions are shown in grey (iREF). (B) Domain structure of full-length DUX4 is shown: N-terminal homeodomains HD1 and HD2, and C-terminal region. Conservation of residues in primates versus human sequences (green curve) C-terminal to residue G153 and sequence alignment of three conserved regions with a disorder value lower than 0.5 (red curve). Residue numbering from UniProt: Q9UBX2. Two helical regions are predicted within the C-terminal region, the first one (cyan helices) and the second one (salmon helix) both containing the amphipathic “ΦXXΦΦ” motif (Φ, bulky hydrophobic amino acid; X, any amino acid) found in several transcription factors reported to interact with KIX (Goto et al., 2002; Radhakrishnan et al., 1997; Wang et al., 2012). The position of the 9aaTAD (blue letters) and KBM (KIX binding motif; red letters) sequences are indicated by black bars. (C) Modelled interactions of the human KIX domain (PDB: 2LXT) with DUX4 9aaTAD (cyan) and KBM (salmon). (D) Replicate wells of HEK-GripTite 293 MSR cells were transfected with either wildtype DUX4 (DUX4 WT) or KIX-binding motif mutant DUX4 (DUX4-KBM mut) both tagged with HA, and with either LACZ or MED15 both tagged with V5. The cell lysates were precipitated for HA-tag. Immunoblots are shown for V5 (above) and HA (below) antibodies. See also Figures S7–S9 and Video S5.

DUX4 has been suggested to function as a pioneer factor (Choi et al., 2016; Hendrickson et al., 2017; Whiddon et al., 2017), given its ability to bind MaLR-enriched condensed chromatin loci and to recruit H3K27 acetyltransferase EP300 leading to locus activation (Choi et al., 2016). We asked whether DUX4 interacts with other proteins that could be related to its ability to accomplish genome-wide transcriptional changes. To this end, we utilized the MAC-tag affinity purification mass spectrometry (AP-MS) method to identify DUX4 protein-protein interactome. As a negative control we used GFP with nuclear localization signal in the same plasmid backbone as DUX4. MAC-tag allows identification of both stable (AP-MS) and dynamic (BioID-MS) protein-protein interactions gathered over the course of 20 h (Liu et al., 2018; Varjosalo et al., 2013). We identified 43 stable AP-MS and 158 transient BioID-MS high-confidence (BFDR < 0.05) DUX4 interactions, including the previously shown DUX4 interaction partners EP300 and cAMP-response element-binding protein (CREB)-binding protein (CBP) (Choi et al., 2016) (Figure S7 and Table S4, including the protein interactions of DUX4 and the negative control). Comparison of our list of DUX4-interacting proteins to the protein complex database (CORUM) yielded significant overrepresentation of the SWI/SNF chromatin remodeling complex, NSL and NuA4 histone acetyltransferase complex, SRCAP histone exchanging complex, and the Core Mediator complex, (FDR < 0.05, Fisher’s exact test; Figure S7). In comparison to the protein-protein interactions of 110 transcription factors that were used as baits in the MAC-tag method (Göös et al., 2021), DUX4 stands out as a notable binding partner of the Mediator complex (Figure 4A). Indeed, out of the 26 known Mediator complex proteins, DUX4 interacted with 16. The majority of the DUX4 protein interactors, including the MED complex proteins, are expressed in human oocytes and pre-implantation embryos (Figure S8). The mammalian Mediator is a transcription coactivator that transduces regulatory signals from transcription factors to RNA polymerase II (Chen et al., 2021). It thus mediates interactions between context-dependent transcription factors, enhancers, and promoters (Soutourina, 2018). Mediator subunit 15 (MED15) was observed as a stable and transient DUX4 protein interactor, suggesting that DUX4 can potentially accomplish some of its suggested functions through interactions with MED15.

To elucidate the functional mechanism of DUX4, we next aimed to identify the protein domain of DUX4 that mediates the interaction with MED15. The DUX4 N-terminal DNA-binding homeodomains are followed by an intrinsically disordered region with three regions of predicted low disorder that are conserved in primates. Within these regions, two predicted amphipathic helices contain a nine amino acid transactivation domain (9aaTAD (Mitsuhashi et al., 2018)), which is also present in another EGA gene, LEUTX (Katayama et al., 2018), and a motif known to recruit the KIX domain (Piskacek et al., 2016) of the CBP (Choi et al., 2016) (Figure 4B). DUX4 has previously been shown to interact with EP300/CBP through its C-terminus (Choi et al., 2016). Indeed, the deletion of the last 98 amino acids from the full-length DUX4 C-terminus abolished the ability of DUX4 to interact with either EP300 or CBP (Choi et al., 2016). The DUX4 C-terminus also has been shown to have a dominant negative activity to full-length DUX4 as shown by co-transfection of the full-length DUX4 and C-terminus of DUX4 leading to inhibition of DUX4-induced expression of its well-known target gene, *ZSCAN4* (Choi et al., 2016). This suggested that DUX4 C-terminus competes with the full-length DUX4 for EP300/CBP. We modeled the 9aaTAD peptide 371GLLLDELLA379 and the KIX binding motif (KBM) 416EYRALL421 into the MLL and pKID/c-Myb site of the ternary complex NMR structure of human KIX from CBP (Bruschweiler et al., 2013) (PDB: 2LXT) (Figure 4C and Data S1). The hydrophobic residues of 9aaTAD and KBM complement well what is seen in the KIX:MLL:pKID complex. Indeed, experimental tight binding (Figures S9A–S9C) was detected for peptides overlapping the 9aaTAD (K_d_≈ 0.2 μM) and KBM (K_d_≈ 0.6 μM) sequences of DUX4 to KIX domain, and for KBM binding in the presence of 9aaTAD (K_d_≈ 1.1 μM). MED15 contains a KIX domain (Thakur et al., 2014) and thus, we speculated that the DUX4 C-terminal KIX binding motif 416EYRALL421 recruits the KIX domain of MED15. To test this, we cloned DUX4 without the C-terminal KBM motif (DUX4-KBM mutant) and performed a co-immunoprecipitation. While the V5-tagged MED15 was precipitated with the HA-tagged wildtype DUX4, no interactions were found in the presence of the DUX4-KBM mutant (Figure 4D). In summary, our analyses suggest that the 6 amino acid KBM at the end of the DUX4 C-terminus mediates interaction with MED15, alluding to DUX4 having all the attributes needed for rapid target activation.

We observed prominent DUX4 immunofluorescence signal in the cytoplasm of the human early embryos (Figures 2B and 2C). We thus asked whether the homeodomain1-linker-homeodomain2 structure would be stabile as a unit without bound DNA and subjected the crystal structure of DUX4 (PDB: 6E8C (Lee et al., 2018), Data S2) to molecular dynamics simulations. Ten residues, highly conserved in primates, formed two interacting clusters (Figures S9D and S9E) stabilizing both domains even in the absence of DNA (Videos S5A and S5B). While predominantly the charge-charge interactions hold the two homeodomains together (Figures S9F–S9I), the intermediate linker loop imparts flexibility, which could be vital to accommodate DNA once DUX4 enters the nucleus and locates its binding motif. Indeed, the double homeodomain without DNA opened dramatically, by over 38 Å, and the stabile open conformation would be suited to initial interactions with DNA and be consistent with the proposed two-step clamp-like binding mechanism (Dong et al., 2018).

Video S5. Simulations of DUX4 structure (A) 360° view of last sampled conformation of DNA-free DUX4 (blue) from the 100 ns simulation superposed on the DNA-bound DUX4 crystal structure (red and grey) (HD1-HD2-comparison.mp4).
(B) Molecular dynamics simulation (100 ns) of DUX4 HD1-HD2 without bound DNA (HD1-HD2.mp4).
Related to Figures 4 and S9.

## Discussion

In agreement with previous studies, our findings highlight *DUX4* as a transcriptome and chromatin modifier (De Iaco et al., 2017; Geng et al., 2012; Hendrickson et al., 2017; Liu et al., 2019; Whiddon et al., 2017), enriched at the earliest stages of human embryo development. Although transcription from LTR-containing repeat loci is well established in mammalian early embryos, implications of these loci have only recently been clarified, for instance, as *cis*-regulatory elements during embryonic lineage specification (Pontis et al., 2019; Todd et al., 2019). Our results reveal numerous *DUX4*-activated ERVL-MaLR regions that overlap with bidirectionally transcribed putative enhancers. We have validated the enhancer activity of two separate regions that can activate *ZSCAN4* and *KHDC1P1*. A CRISPR activation assay was successful with both the VP16 *trans* activator domain-containing construct (Weltner et al., 2018) and the construct where DUX4 C-terminus was fused with dCas9, elucidating DUX4 as a robust enhancer activator (Choi et al., 2016; Hilton et al., 2015). Epigenetic pre-patterning of developmental gene expression has been shown to occur in Zebrafish prior to EGA (Lindeman et al., 2011). Recent evidence also indicates that human embryonic genome undergoes priming that involves the acquisition of a globally permissive chromatin state before major EGA (Xia et al., 2019). Of interest, distal candidate *cis*-regulatory elements are highly accessible in 4-cell stage embryos and may functions as enhancers (Xia et al., 2019). Moreover, recent data also imply that evolutionary young TE elements expressed in the early human embryo can serve as enhancers, also for the genes that are required later in development (Pontis et al., 2019).

siRNA-mediated knockdown of DUX4 in human triploid zygotes did not lead to embryonic arrest by the third day of development, in agreement with what has been shown for *DUX4* knockout mouse (Chen and Zhang, 2019; De Iaco et al., 2017). The siDUX4 blastomeres exhibited minor downregulation of the EGA transcriptome with several retained maternal genes. Maternal mRNA clearance takes place in at least two phases, during oocyte maturation and early embryo development (Vastenhouw et al., 2019), thus before and after EGA, respectively. Recent findings indicate that maternal mRNAs in human oocytes can be clustered based on their degradation rate, suggesting selective mRNA clearance during human maternal-to-zygotic transitions (Sha et al., 2020). Intriguingly, the clearance of a subset of maternal mRNAs was dependent on EGA (Sha et al., 2020). It remains to be elucidated whether *DUX4* directly participates in the clearance of maternal mRNAs and if *DUX4* is required for human embryo development. *DUX4* was recently suggested to play a central role in the regulation of ‘maternally biased genes’ at the 4- to 8-cell stage in a study that investigated parent-of-origin effects in biparental and uniparental human early embryos (Leng et al., 2019). While the *DUX4* binding motif was identified as the most enriched motif for maternally biased genes, many of the putative *DUX4* targets were also involved in a transcriptional regulatory network, indicating that they could also be regulated by other factors, such as *DUXA* and *NANOG* (Leng et al., 2019). In agreement with these analyses, we anticipate that factors other than *DUX4* also function as early regulators of EGA and may compensate for the reduced *DUX4* activity in the siDUX4 embryos.

In addition to several chromatin modifiers, our DUX4 protein interactome analysis revealed contacts with RNA-binding proteins and mRNA splicing proteins (Ansseau et al., 2016). Further studies are required to elucidate whether cytoplasmic DUX4 protein interactions relate to the observed DUX4 protein localization in the cytoplasm of early embryos, and whether they are functionally important. DUX4 has previously been shown to recruit EP300/CBP (Choi et al., 2016). We revealed that the DUX4 C-terminal KIX-binding motif recruits the MED15 protein. This suggests that in addition to recruiting acetyltransferase EP300 and CBP, DUX4 also directly interacts with MED15, most likely associated with DUX4-induced transcription initiation. In conclusion, we characterize the dynamics of DUX4 RNA and protein expression in human zygotes and embryos and elucidate its potential functions in EGA. Our results expand the information about DUX4 as a multifunctional factor that regulates both the coding and non-coding genome.

### Limitations of the study

We note that there are a few limitations to our study. Although we were able to achieve statistical significance for differentially expressed genes in the *DUX4* knockdown experiment, the overall number of blastomeres included in the study was low. We also observed heterogeneity in the expression of genes within siControl and siDUX4 cells potentially due to the use of 3PN embryos. Another possible cause for heterogeneity in gene expression among the siDUX4 embryos is the timing of the microinjection with respect to zygotic enrichment of *DUX4*. The question whether *DUX4* is an essential transcription factor in early human development remains to be resolved. Future studies with a higher number of zygotes and culturing embryos up to the blastocyst stage or 14 days of development following knockdown would provide a broad picture of the role of *DUX4* in human development. Additionally, our study had technological limitations. It is currently not feasible to perform NET-CAGE in single cells in oocytes and embryos, owing to the large number of cells required for the library preparation (Hirabayashi et al., 2019). Therefore, the number of transcribed enhancers that are functionally active in human embryos is yet to be determined.

## STAR★Methods

### Key resources table

**Table:** 

REAGENT or RESOURCE	SOURCE	IDENTIFIER
**Antibodies**		

Rabbit anti-DUX4	Abcam	Cat# ab124699, clone E5-5; RRID: AB_10973363
Donkey Anti-Rabbit IgG (H+L) (Alexa Fluor 488)	Thermo Fisher Scientific	Cat# A-21206; RRID: AB_2535792
Donkey Anti-Rabbit IgG (H+L) (Alexa Fluor 594	Thermo Fisher Scientific	Cat# A-21207; RRID: AB_141637
Anti-HA Tag Antibody	Biolegend	Cat#PRB-101C
Anti-alpha Tubulin antibody	Abcam	Cat#ab7291

**Bacterial and virus strains**		

*E. coli* BL21 DE3 cells	New England BioLabs	Cat# C2527I

**Chemicals, peptides, and recombinant proteins**		

Tween 20	Fisher Scientific	Cat# BP337-100
Triton X-100	Fisher Scientific	Cat# BP151-100
Ultravision protein Block solution	Thermo Fisher Scientific	Cat# TA-060-PBQ
DAPI (4',6-Diamidino-2-Phenylindole, Dilactate)		Cat# D3571; RRID: AB_2307445
Geltrex LDEV-Free, hESC-Qualified, Reduced Growth Factor Basement Membrane Matrix	Thermo Fisher Scientific	Cat# A1413302
Essential 8 Medium	Thermo Fisher Scientific	Cat# A1517001
UltraPure 0.5M EDTA, Ph 8.0	Thermo Fisher Scientific	Cat# 15575020
StemProAccutase Cell Dissociation Reagent	Thermo Fisher Scientific	Cat# A1110501
TrypLE Express Enzyme	Thermo Fisher Scientific	Cat# 12604-021
Fetal bovine serum, qualified, Brazil	Thermo Fisher Scientific	Cat# 10270106
Rock inhibitor Y27632	Selleckhem	Cat# S1049
Puromycin dihydrochloride	Thermo Fisher Scientific	Cat# A11138-03
Doxycycline hyclate	Sigma Aldrich	Cat# D9891 Lot: #017M4011V
IGEPAL CA-630	Sigma Aldrich	Cat# 18896
Phusion PCR Master mix	Thermo Fisher Scientific	Cat# F531L
FuGENE HD transfection reagent	Promega	Cat# E2311
RiboLock	Thermo Fisher Scientific	Cat# EO0382
MMLV-RTase	Promega	Cat# M1701
DUX4 9aaTAD peptide for binding analysis: CGLLLDELLASPEFLQQ	GenScript	N/A
DUX4 KBM peptide for binding analysis: EEEYRALLEE	GenScript	N/A
Histrap HP column (1 ml)	GE Healthcare	Cat# 17524701
Resource Q column (1 ml)	GE Healthcare	Cat# 17117701
Superdex 75 10/300 GL column	GE Healthcare	Cat# 29148721
CelluSep dialysis membrane, MWCO 6-8K	Membrane Filtration Products, Inc.	Cat# 132650
Amicon Ultra-4 centrifugal filter, MWCO 3K	Merck Millipore	Cat# UFC800324
ZYM-5025 autoinduction medium	(Studier, 2005)	N/A
Strep-Tactin®Sepharose® 50% suspension	IBA life sciences	Cat# 2-1201-010
Gateway™ LR Clonase™ Enzyme Mix	Life Technologies	Cat# 11791043
FuGENE® 6 Transfection Reagent	Promega	Cat# E2691
Hygromycin B	Life Technologies	Cat# 10687010
Biotin	Thermo Scientific	Cat# 29129
Benzonase®Nuclease	Santa Cruz Biotechnology	Cat# sc-202391
Tetracycline hydrochloride	Sigma-Aldrich	Cat# T3383
Alpha-amanitin	FUJIFILM Wako Pure Chemical	Cat# 010-22961
cOmplete Protease Inhibitor Cocktail	Sigma-Aldrich	Cat# 4693116001
SUPERaseIN RNase Inhibitor	Thermo Fisher Scientific	Cat# AM2696
QIAzol Lysis Reagent	QIAGEN	Cat# 79306
Tergitol solution Type NP-40, 70% in H2O	Sigma-Aldrich	Cat# NP40S-100ML
1mol/l-HEPES Buffer Solution	Nacalai tesque	Cat# 17557-94
(+/-)-Dithiothreitol	FUJIFILM Wako Pure Chemical	Cat# 048-29224
Urea	FUJIFILM Wako Pure Chemical	Cat# 219-00175
Ethylenediamine-N,N,N',N'-tetraacetic Acid Diammonium Salt	FUJIFILM Wako Pure Chemical	Cat# 346-01971
Ambion DNase I (RNase-free)	Thermo Fisher Scientific	Cat# AM2224
Rnase Free Dnase set	QIAGEN	Cat# 79254
TRIzol Reagent	Thermo Fisher Scientific	Cat# 15596018

**Critical commercial assays**		

Neon transfection system 100 μl kit	Thermo Fisher Scientific	Cat# MPK10096
Nextera DNA sample preparation kit, Illumina	Illumina	Cat# FC-121-1030
Nextera DNA Library Prep	Illumina	Cat# 15028212, Batch 20241118
NextSeq 500/550 High Output kit v2.5 (75 cycles)	Illumina	Cat# 20024906
GeneJET PCR purification Kit	Thermo Fisher Scientific	Cat# K0701
NucleoSpin RNA	Macherey Nagel	Cat# 740955.250
HOT FIREpol qPCR Master Mix	Solis Biodyne	Cat# 08-25-00020
Monolith NT(TM) His-Tag Labeling Kit RED-tris-NTA (1st generation)	NanoTemper Technologies	Cat# MO-L018
NucleoSpin Plasmid Easypure	Macherey-Nagel	Cat#740727
miRNeasy Mini kit	QIAGEN	Cat# 217004

**Deposited data**		

KIX-9aaTAD-KBM.pdb	This paper	Data S1
DUX4_HD1-HD2.pdb	This paper	Data S2
HD1-HD2-comparison.mp4	This paper	Video S5A
HD1-HD2.mp4	This paper	Video S5B
ATAC-seq, CAGE/NET-CAGE and bulk STRT datasets	This paper	Gene Expression Omnibus GSE171803
Sequences of the cloned transcripts	This paper	European Nucleotide Archive LR694082-LR694089

**Experimental models: Cell lines**		

Human: DUX4-TetON human ES cell: WA01	This paper	N/A
Human: DUX4-TetON human ES cell: WA09	This paper	N/A
Human: HEK-293	ATCC	Cat# CRL-1573
Human: Flp-In™ T-REx™ 293 cells	Thermo Fisher Scientific	Cat# R78007

**Oligonucleotides**		

qPCR, *DUX4* Forward	AGGAAGAATACCGGGCTCTG	N/A
qPCR, *DUX4* Reverse	AGTCTCTCACCGGGCCTAG	N/A
qPCR, *ZSCAN4* Forward	CCTCCCAGACTTCCCAAGAT	N/A
qPCR, *ZSCAN4* Reverse	TGTTCCAGCCATCTTGTTCA	N/A
qPCR, *TRIM48* Forward	CATCACTGGACTGAGGGACA	N/A
qPCR, *TRIM48* Reverse	TGACTGTTGGCTTCATTGTGA	N/A
qPCR, *KHDC1P1* Forward	CCTGTCGTCACAATCAAGGC	N/A
qPCR, *KHDC1P1* Reverse	TCCACTTATCCTGGAGGCCA	N/A
qPCR, *CYCLOPHILIN G* Forward	TCTTGTCAATGGCCAACAGA	N/A
qPCR, *CYCLOPHILIN G* Reverse	GCCCATCTAAATGAGGAGTT	N/A
DUX4 C-terminus cloning: DUX4 clone Forward	CTGCTCGAGTTCGAAGGCAG CGGCGGGGGCAGGGCGCCC GCGCA	N/A
DUX4 C-terminus cloning: DUX4 clone Reverse	CATGCGGCCGCACTAGTGTCGA CTCTAGAAAGCTCCTCCAGCAG AGCCC	N/A
DUX4 Forward (for MAC tag)	AAAAAGCAGGCTCCACCATGG CCCTCCCGACAC	N/A
DUX4 Reverse (for MAC tag)	AGAAAGCTGGGTCAAGCTCCT CCAGCAGAGCC	N/A
DUX4 KIX-binding mutant clone Reverse (for MAC tag)	AGAAAGCTGGGTCAAGCTCCT CTTCCTCGCTGA GGGGTGCTTC	N/A
ZSCAN4 guide RNA 1 oligo sequence 5' to 3' (Benchling sequence underlined)	GTGGAAAGGACGAAACACCG CTTAGTACATTACCAAAACCG TTTTAGAGCTAGAAATAG	N/A
ZSCAN4 guide RNA 2 oligo sequence 5' to 3' (Benchling sequence underlined)	GTGGAAAGGACGAAACACCG AATGTAATCTCCAATGTTGGG TTTTAGAGCTAGAAATAG	N/A
ZSCAN4 guide RNA 3 oligo sequence 5' to 3' (Benchling sequence underlined)	GTGGAAAGGACGAAACACCG AGGAGGTGATTGGCTCATGAG TTTTAGAGCTAGAAATAG	N/A
ZSCAN4 guide RNA 4 oligo sequence 5' to 3' (Benchling sequence underlined)	GTGGAAAGGACGAAACACCG GGTGGTGCTAAACCATTCAGG TTTTAGAGCTAGAAATAG	N/A
ZSCAN4 guide RNA 5 oligo sequence 5' to 3' (Benchling sequence underlined)	GTGGAAAGGACGAAACACCG CATGATAACTCACTATCGTGG TTTTAGAGCTAGAAATAG	N/A
KHDC1P1 guide RNA 1 oligo sequence 5' to 3' (Benchling sequence underlined)	GTGGAAAGGACGAAACACCG CCCATTGTAGGAGTTGACTAG TTTTAGAGCTAGAAATAG	N/A
KHDC1P1 guide RNA 2 oligo sequence 5' to 3' (Benchling sequence underlined)	GTGGAAAGGACGAAACACCG TCGTGTTGGAATACACTGTGG TTTTAGAGCTAGAAATAG	N/A
KHDC1P1 guide RNA 3 oligo sequence 5' to 3' (Benchling sequence underlined)	GTGGAAAGGACGAAACACCG GAGGGTATGGAGGTGCAGGAG TTTTAGAGCTAGAAATAG	N/A
KHDC1P1 guide RNA 4 0ligo sequence 5' to 3' (Benchling sequence underlined)	GTGGAAAGGACGAAACACCG TTATCTTGGGAAGACCTCCGG TTTTAGAGCTAGAAATAG	N/A
KHDC1P1 guide RNA5 oligo sequence 5' to 3' (Benchling sequence underlined)	GTGGAAAGGACGAAACACCG GGTGGATGTCCAATCCCCGGG TTTTAGAGCTAGAAATAG	N/A

**Recombinant DNA**		

pBASE	(Wang et al., 2008)	N/A
pPB-CAG-rtTA-M2-IN	(Takashima et al., 2014)	Addgene #60612
pB-tight-DUX4-ires-EmGFP-pA-PGK-Puro	This paper	N/A
dCas9-VP192-2A-GFP	(Balboa et al., 2015)	N/A
dCas9-DUX4-C-2A-GFP	This paper	N/A
MAC-tag-C-DUX4 WT	This paper	N/A
MAC-tag-C-DUX4 KBM mutant	This paper	N/A
MAC-tag-C	(Liu et al., 2018)	Addgene #108077
pET100/TOPO vector with synthetic gene coding for human KIX domain of CBP (residues 587-673; Uniprot Q92793)	Invitrogen GeneArt Gene Synthesis, Thermo Scientific; this paper	N/A
pOG44 Flp-Recombinase Expression Vector	Life Technologies	Cat#V600520
Gateway™ pDONR221™	Thermo Scientific	Cat#12536017

**Software and algorithms**		

Benchling CRISPR Guide RNA design		https://www.benchling.com/crispr/
MAFFT	(Katoh and Standley, 2013)	https://mafft.cbrc.jp/alignment/software/
SCRATCH	(Cheng et al., 2005)	http://scratch.proteomics.ics.uci.edu/index.html
RaptorX-Property	(Wang et al., 2016)	http://raptorx.uchicago.edu/StructurePropertyPred/predict/
9aaTAD web server	(Piskacek et al., 2007)	https://www.med.muni.cz/9aaTAD/
PDB (the Protein Data Bank)	(Berman et al., 2000)	https://www.rcsb.org
UniProt database	The UniProt Consortium, 2019	https://www.uniprot.org
Blastp	(Johnson et al., 2008)	https://blast.ncbi.nlm.nih.gov/Blast.cgi?PAGE=Proteins
PyMOL (v 2.4)	Schrödinger LLC	https://pymol.org
Bodil	(Lehtonen et al., 2004)	
Chimera	(Pettersen et al., 2004)	https://www.rbvi.ucsf.edu/chimera
AMBER package (v 18; Case, D.A., 2018)		https://ambermd.org/CiteAmber.php
ff14SB force field	(Maier et al., 2015)	
OL15 force field	(Zgarbova et al., 2015)	
CPPTRAJ	(Roe and Cheatham, 2013)	
VMD	(Humphrey et al., 1996)	https://www.ks.uiuc.edu/Research/vmd/
Particle-mesh Ewald algorithm	(Essmann et al., 1995)	
Explicit TIP3P water molecules	(Jorgensen et al., 1983)	
Proteome Discoverer (v 1.4)	Thermo Fisher Scientific	https://www.thermofisher.com/fi/en/home/industrial/mass-spectrometry/liquid-chromatography-mass-spectrometry-lc-ms/lc-ms-software/multi-omics-data-analysis/proteome-discoverer-software.html
Cytoscape (v 3.6.)	(Shannon et al., 2003)	https://cytoscape.org/
SAINTexpress (v 3.6.3)	(Teo et al., 2014)	http://saint-apms.sourceforge.net/Main.html
XCalibur (v 3.0.63)	Thermo Fisher Scientific	https://www.thermofisher.com/order/catalog/product/OPTON-30965#/OPTON-30965
MOIRAI	(Hasegawa et al., 2014)	http://fantom.gsc.riken.jp/software/
STAR (v 2.5.0a)	(Dobin et al., 2013)	https://github.com/alexdobin/STAR
Cutadapt (v 1.1.8)	(Martin, 2011)	https://cutadapt.readthedocs.io/en/stable/
Bedtools (v 2.27.2)	(Quinlan and Hall, 2010)	http://bedtools.readthedocs.io/en/latest/
Samtools (v 0.1.19)	(Li et al., 2009)	http://www.htslib.org
Decomposition peak identification (DPI)	(Forrest et al., 2014)	https://github.com/hkawaji/dpi1
Bi-directional enhancer identification	(Andersson et al., 2014)	https://github.com/anderssonrobin/enhancers
R (v 3.6.1)	R Core Team	https://www.r-project.org/
edgeR (v 3.16.5)	(McCarthy et al., 2012; Robinson et al., 2010)	https://bioconductor.org/packages/release/bioc/html/edgeR.html
ggplot2 (v 3.3.5)		https://cran.r-project.org/web/packages/ggplot2/index.html
gplots (v 3.1.1)		https://cran.r-project.org/web/packages/gplots/index.html
SAMstrt (v 0.99.0)	(Katayama et al., 2013)	https://github.com/shka/R-SAMstrt
STRTprep3	(Krjutskov et al., 2016a; 2016b)	https://github.com/shka/STRTprep
TopHat (v 2.1.1)	(Kim et al., 2013; Trapnell et al., 2009)	https://ccb.jhu.edu/software/tophat/index.shtml
Bowtie (v 1.1.2.0)	(Langmead et al., 2009)	http://bowtie-bio.sourceforge.net/index.shtml
HOMER	(Heinz et al., 2010)	http://homer.ucsd.edu/homer/
Metascape	(Zhou et al., 2019)	https://metascape.org/gp/index.html#/main/step1
SH800 (v 1.7)	SONY Biotechnology	https://www.sonybiotechnology.com/us/instruments/sh800s-cell-sorter/software/
Imaris (v 9.3)	Bitplane, Oxford Instruments	https://imaris.oxinst.com/versions/9-3

**Other**		

μ-slide 8-well	Ibidi	Cat# 80826
Monolith NT.Automated Capillary Chips	NanoTemper Technologies	Cat# MO-AK002
ÄKTA Pure 25 chromatography system	GE Healthcare	Cat# 29018226
NanoDrop One	Thermo Scientific	Cat# ND-ONE-W
Monolith NT(TM) microscale thermophoresis instrument	NanoTemper Technologies	N/A
PDB:2LXT	(Bruschweiler et al., 2013)	https://www.rcsb.org/structure/2LXT
PDB:6E8C	(Lee et al., 2018)	https://www.rcsb.org/structure/6E8C
G-TL embryo culture medium	Vitrolife	Cat# 10145
Biopsy Medium	Origio	Cat# 10620010
DUX4 protein sequence *Homo sapiens*	UniProt	Q9UBX2
DUXA protein sequence *Homo sapiens*	UniProt	A6NLW8
DUXB protein sequence *Homo sapiens*	UniProt	A0A1W2PPF3
DUX1 protein sequence *Homo sapiens*	UniProt	O43812
DUX3 protein sequence *Homo sapiens*	UniProt	Q96PT4
DUX5 protein sequence *Homo sapiens*	UniProt	Q96PT3
DUX4 protein sequence *Pan troglodytes*	NCBI	XP_024209610.1
DUX4 protein sequence *Gorilla gorilla gorilla*	NCBI	XP_018890005.1
DUX4 protein sequence *Pongo abelii*	NCBI	XP_024097529.1
DUX4 protein sequence *Colobus angolensis palliatus*	NCBI	XP_011811800.1
DUX4 protein sequence *Papio anubis*	NCBI	XP_021788945.1
DUX4 protein sequence *Macaca mulatta*	GenBank	CAL41941.1
DUX4 protein sequence *Piliocolobustephrosceles*	NCBI	XP_026306328.1
DUX4 protein sequence *Rhinopithecusroxellana*	NCBI	XP_010379696.1
DUX4 protein sequence *Cebus capucinus imitator*	NCBI	XP_017356904.1
DUX4 protein sequence *Callithrix jacchus*	NCBI	XP_008989085.1
DUX4 protein sequence *Macaca fascicularis*	NCBI	XP_005583211.2

### Resource availability

#### Lead contact

Further information and reasonable requests for resources and reagents should be directed to and will be fulfilled by the lead contact, Sanna Vuoristo (sanna.vuoristo@helsinki.fi).

#### Materials availability

This study did not generate new unique reagents.

### Experimental model and subject details

Collection and experiments on human oocytes and embryos were approved by the Helsinki University Hospital ethical committee, diary numbers 308/13/03/03/2015 and HUS/1069/2016. Human surplus zygotes and embryos were donated by couples that had undergone infertility treatments at the Reproduction Medicine Unit of the Helsinki University Hospital. The donations were done with an informed consent.

### Method details

#### Human ESC culture

hESC lines H1 (WA01) and H9 (WA09) were maintained on Geltrex, hESC-qualified, reduced growth factor basement membrane matrix-coated tissue culture dishes in Essential 8 culture medium and passaged every three to five days by 3–5-min incubation with 0.5 mM EDTA (all from Thermo Fisher Scientific).

#### Generation of DUX4 TetOn human embryonic stem cells

hESCs were incubated with StemPro Accutase (Thermo Fisher Scientific) until the edges of the colonies started to curl up. The Accutase was aspirated, and the cells were gently detached in cold 5% FBS (Thermo Fisher Scientific) 1×PBS (Corning) and counted. One million cells were centrifuged at 107×g for 5 min and the pellet was transferred into 120 μL of R-buffer containing 1 μg of pB-tight-DUX4-ires-EmGFP-pA-PGK-Puro, 0.5 μg of pBASE (Wang et al., 2008)and 0.5 μg of rtTA-M2-IN plasmids (Takashima et al., 2014). 100 μL of the cell-plasmid suspension was electroporated with two pulses of 1100V, 20 ms pulse width, using Neon Transfection system (Thermo Fischer Scientific). The electroporated cells were plated on Geltrex-coated dishes in Essential 8 medium with 10 μM ROCK inhibitor Y27632 (Selleckhem). The following day, the medium was exchanged with fresh Essential 8 medium without ROCK inhibitor. The cells were selected with Puromycin at 0.3 μg/mL. The *DUX4*TetOn hESC clones were picked manually on Geltrex-coated 96-well plates, expanded, and selected again with Puromycin. Appearance of the EmGFP reporter protein was tested using Doxycycline at concentrations ranging from 0.2 μg/mL to 1.0 μg/mL and detected using an EVOS FL Cell imaging system (Thermo Fisher Scientific). For the experiments presented in this paper, the *DUX4*TetOn hESCs have been treated with 1μg/ml of Doxycycline for 4 h STRT-RNA seq, ATAC-seq, NET-CAGE, prior to subsequent analyses.

#### Immunocytochemistry of human ESC

Cells were fixed with 3.8% PFA, washed three times, permeabilised in 0.5% (v/v) Triton X-100 in PBS for 7 min, and washed with washing buffer (0.1% (v/v) Tween20 in PBS). The samples were incubated with ProteinBlock (Thermo Fisher Scientific) at room temperature for 10 min to prevent unspecific binding of primary antibody. Primary antibody (rabbit MAb anti-DUX4, clone E5-5, Abcam) was diluted 1:300 in washing buffer and incubated at 4°C overnight. After washings, fluorescence-conjugated secondary antibody (anti rabbit 594, A-21207; Thermo Fisher Scientific) was diluted 1:1000 in washing buffer and incubated at room temperature for 20 min. Nuclei were counterstained with DAPI 1:1000 in washing buffer. The images were captured with an Evos FL Cell Imaging system using 10× and 20× Plan Achromatic objectives.

#### ATAC-sequencing library preparation

The ATAC-sequencing libraries were prepared as in (Buenrostro et al., 2015). For preparation of the ATAC-seq libraries, the cells were detached by a 5-min TrypLE incubation, washed in cold 5% FBS-PBS, and separated in flow cytometry based on EmGFP expression, which indicates DUX4 expression. 5×10^4^ EmGFP (−) and EmGFP (+) *DUX4*TetOn-hESCs (H1 clone 2, H1 clone 8, H9 clone 3 and H9 clone 4) were centrifuged at 500×g for 5 min. The pellets were washed in cold 1× PBS by centrifugation at 500×g for 5min. Each cell pellet was lysed in 50 μL of cold lysis buffer (10 mM Tris-HCl, pH 7.4, 10 mM NaCl, 3 mM MgCl_2_, and 0.1% IGEPAL CA-630) and centrifuged at 500×g at 4°C for 10 min. The pellet was then resuspended in the transposase reaction mix (2.5 μL of transposase in TD buffer (Nextera DNA library preparation kit, Illumina) and incubated at 37°C for 30 min. The reactions were purified through columns and eluted in 20 μL. After addition of the barcode oligos the DNA samples were amplified for 12 cycles (98°C for 10 s, 63°C for 30 s and 72°C for 60 s) in Phusion PCR master mix (Thermo Fisher Scientific). The PCR products were purified through the columns and eluted in 20 μL. All ATAC-seq libraries were sequenced in single-end mode on an Illumina NextSeq 550 platform using the 75 cycles High Output Kit (v2.5).

#### ATAC-sequencing data analysis

Bcl files were converted and demultiplexed to fastq using the bcl2fastq program. STAR (Dobin et al., 2013) was used to index the human reference genome (hg19), obtained from UCSC(Kent et al., 2002), and align the resulting fastq files. The resulting bam files with the mapped reads were then converted to tag directories with subsequent peaks calling using the HOMER suit of programs (Heinz et al., 2010). HOMER was also employed for counting the reads in the identified peak regions. Peak calling was performed twice, the first time with the default setting of HOMER and the second time with flag -style histone to identify broad peaks. Peaks were merged using the mergePeaks function in HOMER. Reads mapping to the merged peaks were counted using the annotatePeaks.pl function the -noadj flag. The raw tag counts from the peaks were then imported to R/Bioconductor and differential peak analysis between EmGFP (−) and EmGFP (+) *DUX4*TetOn-hESCs with four biological replicates each was performed using the edgeR package and its general linear models pipeline. Peaks with an FDR-adjusted p value < 0.05 were termed as control or *DUX4*-activated.

#### Library preparation, sequencing and read-alignment for CAGE-based data

Nascent RNA from flash-frozen cells was isolated as described by Hirabayashi et al., (2019) (Hirabayashi et al., 2019) with the following modifications: (i) DNase I enzyme (50 Units, Thermo Fisher Scientific) was used to prepare the DNase I solution (50 μL), (ii) the samples were incubated for up to 1 h at 37°C while being pipetted up and down several times every 10 min, and (iii) RNA quality was measured using a TapeStation4200 (Agilent). CAGE-based libraries were generated according to the no-amplification non-tagging CAGE libraries for Illumina next-generation sequencers (nAnT-iCAGE) protocol(Murata et al., 2014). All CAGE-based libraries were sequenced in single-read mode on an Illumina NextSeq500 platform. Reads were split by barcode using the MOIRAI (Hasegawa et al., 2014) package. Cutadapt v 1.1.8 (Martin, 2011) (http://code.google.com/p/cutadapt/) was used to trim reads to 73 bp and remove reads below base quality 33 and ‘N’ bases. Reads aligning to ribosomal RNA sequences (GenBank U13369.1) were removed using the rRNAdust script within the MOIRAI package. The resulting reads were aligned to the human genome (hg19) using STAR v 2.5.0a (Dobin et al., 2013) with Gencode v27lift37 (“comprehensive”) (Harrow et al., 2012) as the reference gene model. Mapping was performed with the following parameters: --runThreadN 12 --outSAMtype BAM SortedByCoordinate --out FilterMultimapNmax 1. Following alignment, the re-sequenced replicates were merged using the Picard Toolkit v 2.0.1 with the MergeSamFiles program (Broad Institute, Picard Toolkit, 2018. http://broadinstitute.github.io/picard) resulting in two control (dox -) and two *DUX4*-expressing samples each for CAGE and NET-CAGE. BAM files were indexed using Samtools v 0.1.19 (Li et al., 2009)converted to bed files using BEDTools v 2.27.2(Quinlan and Hall, 2010). Transcription start sites (TSSs) were identified for all CAGE and NET-CAGE samples according to http://fantom.gsc.riken.jp/5/sstar/Protocols:HeliScopeCAGE_read_alignment. The TSSs bed files were converted to strand specific bedGraph files and subsequently to bigWig files using the UCSC software bedGraphtobigWig.

#### Identification of transcribed promoters and enhancers

To identify promoter and enhancer regions, TSSs that mapped close to each other on the same strand were grouped into clusters. This was performed using decomposition peak identification (Forrest et al., 2014)

(https://github.com/hkawaji/dpi1/blob/master/identify_tss_peaks.sh) with default parameters but without the decomposition composition parameter. TSS clusters with at least three supporting CAGE tags were retained and used as input to identify bidirectionally transcribed enhancers.

(https://github.com/anderssonrobin/enhancers/blob/master/scripts/bidir_enhancers).

Promoter TSS clusters that were defined as those that did not overlap enhancers and mapped to +/− 300bp of the 5′-end of GENCODE v 27 transcripts. For differential expression (DE) analysis between control and DUX4 expressing hESC, we first counted the TSSs mapping to promoters and enhancers. Next, coverage at single-base-pair resolution was calculated with BEDTools v 2.27.2 (http://bedtools.readthedocs.io/en/latest/) using only the 5′ ends of the reads. The resulting forward and reverse bedGraph files were then converted into bigWig files using the UCSC software bedGraphtobigWig. Counting was performed using in a strand-specific manner using UCSC software bigWigAverageOverBed. Normalization and DE was performed using egdeR v3.26.8 (McCarthy et al., 2012; Robinson et al., 2010).

(https://bioconductor.org/packages/release/bioc/html/edgeR.html). Promoter counts were normalized using calcNormFactors function with relative log expression, and counts were converted to log2 counts per million (CPM). A prior count of 0.25 was added to the raw counts. For enhancers forward and reverse counts were summed up. The counts were normalized using the same normalization factors as generated for promoters. Promoters (log2 CPM >−2.0) and enhancers (log2 CPM >−3.5) expressed in at least one library were retained. DE was performed between four controls (dox -) and four *DUX4*-expressing (dox +) expressing samples with Benjamini–Hochberg false discovery rate (FDR) correction.

#### Repeat element analysis

RepeatMasker table was downloaded from UCSC table browser (http://hgdownload.soe.ucsc.edu/goldenPath/hg19/database/rmsk.txt.gz) and converted to BED format. The length of ATAC-seq peaks and NET-CAGE promoters and enhancers was extended from the center such that all regions are 600 bp long. Random background regions were generated using the bedtools random function such that the number and length of the background regions were the same as the region of interest. Repeat elements overlapping the regions of interest or background were identified using intersectBed with parameter -wo from BEDTools v2.27.2 (Quinlan and Hall, 2010). If more than one repeat element overlapped the regions, then the element with the longest overlap (base pairs) was chosen. The frequency of repeat elements overlapping the regions of interest and background was calculated. The log2 ratio of frequencies (region of interest/background region) has been shown.

#### Bulk RNA-sequencing of FACS sorted cells using the STRT method

TetOn-DUX4 hESCs either with or without doxicycline treatment were washed with PBS and incubated with TrypLE for 5 min, detached, and suspended into cold FACS buffer (5% FBS in PBS). The cell suspension was filtered through Cell strainers to remove any cell clumps and centrifuged at 800 rpm for 5 min. The cell pellets from Dox (+) and Dox (−) cultures were suspended in the cold FACS buffer and placed on ice. EmGFP (−) cells from the Dox (−) and EmGFP (+) cells from the Dox (+) suspension were sorted into cold FACS buffer using a Sony SH800Z Cell Sorter with blue laser (488) and 100 μm nozzle. Total RNA was isolated from FAC-sorted DUX4-TetOnhES cells using the RNAqueous Total RNA Isolation Kit (AM1912; Thermo Fisher Scientific). 20 ng of total RNA from each sample was used for library preparations. The libraries were prepared using the STRT method as above, with minor modifications. Briefly, RNA samples were placed in a 48-well plate in which a universal primer, template-switching oligos, and a well-specific 8 bp barcode sequence were added to each well (Krjutskov et al., 2016a). The synthesized cDNAs from the samples were then pooled into one library and amplified by single-primer PCR with the universal primer sequence. The resulting amplified library was then sequenced using an Illumina NextSeq500 instrument. Alignment of raw reads to the hg19 reference genome, normalization and DE was performed as per the STRTprep pipeline (Krjutskov et al., 2016a).

#### cDNA cloning of previously unannotated genes

A cDNA library was prepared from a single human 4-cell embryo according to the protocol by Tang et al., (2010) (Tang et al., 2010) and used for cloning of putative transcripts. Transcripts were amplified using Phusion High-Fidelity DNA polymerase (New England Biolabs) according to manufacturer's instructions. The previously unannotated *KHDC1P1*, putative *RETT-FINGER TYPE E3 UBIQUITIN LIGASE*, and putative *RING-FINGER DOMAIN PROTEIN* encoding genes were amplified using touchdown PCR: 98°C for 30 s; 24 cycles of 98°C for 10 s, annealing for 30 s, temperature decreasing from 63°C to 56°C, 1°C/3 cycles, 72°C for 30 s; 16 cycles of 98°C for 10 s, 55°C for 30 s, 72°C for 30 s; final extension 72°C for 10 min. All PCR products were cloned into pCR4Blunt-TOPO vector using the Zero Blunt TOPO PCR Cloning kit (Invitrogen) and sequences were verified by Sanger sequencing (Eurofins Genomics). Clone sequences are available from the ENA browser at http://www.ebi.ac.uk/ena/data/view/LR694082-LR694089.

#### *KHDC1P1* and *ZSCAN4* enhancer validation

Putative *KHDC1P1* and *ZSCAN4* enhancer regions were predicted from *DUX4*TetOn hESC NET-CAGE dataset. The guide RNAs targeting the each of the putative enhancers were designed using the Benchling CRISPR tool (https://benchling.com), targeting them +/−200 base pairs of the putative enhancer midpoint. Guide sequences were selected according to their on- and off-target score and position. Guide RNA oligos are shown in key resources table. Guide RNA transcriptional units (gRNA-PCR) were prepared by PCR amplification with Phusion polymerase (Thermo Fisher), using as template U6 promoter and terminator PCR products amplified from pX335 together with a guide RNA sequence-containing oligo to bridge the gap. The oligos for guide RNA transcriptional units are as in (Balboa et al., 2015). PCR reaction contained 50 pmol forward and reverse primers, 2 pmol guide oligo, 5 ng U6 promoter and 5 ng terminator PCR products in a total reaction volume of 100μL. The PCR reaction program was 98°C/10sec, 56°C/30sec, 72°C/12sec for 35 cycles. Amplified gRNA-PCRs were purified and transfected to HEK293 cells as described in (Balboa et al., 2015).

#### HEK cell transfections

HEK 293 cells were seeded on tissue culture treated 24-well plates one day prior to transfection (5 × 10^4^ cells/well). Cells were transfected using FuGENE HD transfection reagent (Promega) in fibroblast culture medium with 500 ng of either dCas9-DUX4-C or dCas9VP192 transactivator encoding plasmid and 200 ng of guide RNA-PCR product or TdTomato guide RNA plasmid. Cells were cultured for 72 h post-transfection, after which samples were collected for qRT-PCR.

#### RNA isolation, reverse transcription and quantitative real-time PCR from DUX4 TetOn hESCs and HEK293 cells

Total RNA was isolated using NucleoSpin RNA kit (Macherey Nagel). 1μg of RNA was reverse transcribed by MMLV-RTase with oligo dT, dNTPs, and Ribolock in MMLV-RTase buffer (Thermo Fisher Scientific). 5× HOT FIREPol qPCR Mix (Solis Biodyne) was used to measure relative mRNA levels with LightCycler (Roche). The ΔΔCT method was followed to quantify the relative gene expression where *CYCLOPHILIN G* (*PPIG*) was used as endogenous control. Relative expression of each gene was normalized to the expression without doxicycline treatment. The primer sequences are listed in the key resources table. Cells transfected with either dCas9-DUX4-C or dCas9-VP192 transactivator together with TdTomato targeting guide plasmid and were used as controls.

#### Data analyses on published single-cell tagged reverse transcription (STRT) data from human oocytes and embryos

We analysed single cell RNA-sequencing data from Tohonen et al., (2015) (Tohonen et al., 2015) for MII oocytes (n = 20), zygotes (n = 59), 2-cell (n = 4), 4-cell (n = 15) and 8-cell (n = 14) embryos. The expression of DUX4 is elusive due to a high number of identical or nearly identical copies present in the human genome. To avoid this complexity, we directly mapped STRT raw reads to a single copy of the DUX4 sequence (ENST00000565211.1 ± 200bp, corresponding to the genomic region of Chr4:190173575-190176045 in GRCh38/hg38) using bwamem ver.0.7.15-r1140(Li and Durbin, 2009). STRT data of the early human embryo obtained from Tohonen et al., (2015) (Tohonen et al., 2015) were overlapped with TFEs using the intersectBed function from BEDTools (Quinlan and Hall, 2010) (v2.27.1).

#### Immunocytochemistry of human embryos

For characterization and quantitation of the DUX4 protein diploid zygotes (n = 3) and embryos (2-cell, n = 3; 4-cell, n = 4; 8-cell, n = 2 were fixed in 3.8 % PFA at room temperature for 15 min, washed three times in washing buffer (0.1% Tween 20 in PBS), and permeabilised in 0.5% Triton X-100 in PBS at room temperature for 15 min and washed once. Unspecific primary antibody binding was blocked with ProteinBlock (Thermo Fisher Scientific) by incubation at room temperature for 10 min. Primary antibody (rabbit MAb anti-DUX4, clone E5-5, Abcam) was diluted 1:300 in washing buffer and incubated at 4°C overnight. After three washes, the embryos were incubated in the secondary antibody (anti-rabbit Alexa 488, A-21206; Thermo Fisher Scientific) diluted 1:500 in washing buffer (as above) at room temperature for 2 h. After three washes, nuclei were counterstained with DAPI 1:500 in washing buffer.

#### Confocal microscopy and image analysis of embryos

Human embryos were imaged in washing buffer on Ibidi 8-well μ slides with a Leica TCS SP8 confocal laser scanning microscope (Leica Microsystems, Mannheim, Germany) using Leica HC PL APO CS2 40×/1.10NA and Leica HC PL APO CS2 63×/1.20NA water objectives. Confocal images were processed using Fiji (http://fiji.sc). For the data presented in Figure 2 B and 2C, images were smoothened using a Gaussian filter (radius = 1-pixel kernel). For the quantification of the DUX4 intensity in the nucleus (Figure 1D), the DAPI channel was denoised using a rolling ball (radius = 100). The images were smoothened in 3D using a Gaussian filter (radius = 2-pixel kernel) and cell nuclei were segmented. The segmented regions were used to measure average pixel intensity per nucleus in each cell in the DUX4 channel. DUX4 intensity in the nucleus was normalized to intensity of the corresponding cytoplasmic DUX4 staining in the single representative plane. 3D renderings were obtained with the Imaris Software v9.3 (Bitplane, Oxford Instruments).

#### Culture and microinjection of human embryos

Human triploid zygotes were warmed using a Gems Warming Set (GeneaBiomedx) and cultured in G-TL medium (Vitrolife) under oil, in 6% O_2_ and 6% CO_2_ at 37°C. Twelve μl of either 20 μM scrambled control siRNA (AM4611, Thermo Fisher Scientific) or *DUX4* targeting siRNA (cat.# 4457308, Thermo Fisher Scientific) were diluted in nucleotide-free H_2_O and centrifuged at maximum speed at 4°C for 10 min. The zygotes were microinjected using FemtoJet 4i microinjector (Eppendorf) and placed in G-TL medium in a Geri dish for time-lapse imaging (Geri incubator, GeneaBiomedx, Australia). To confirm that *DUX4* targeting siRNA efficiently reduced DUX4, control siRNA (siControl, n = 4) or *DUX4* targeting siRNA (siDUX4, n = 5) microinjected zygotes were immunostained for DUX4 and imaged using confocal microscopy.

#### STRT RNA-seq in human pre-implantation embryos

For the *DUX4* knockdown experiment, zygotes microinjected with either siControl or si*DUX4* were cultured for 48 h post microinjections, until 8-cell to morula stage. A part of the zona pellucida was removed using laser microdissection. After release from the zona pellucida, each embryo was incubated in Ca^2+^/Mg^2+^-free Biopsy Medium (Origio) at 37°C on a heated stage for separation of the cells. Individual cells were briefly rinsed in Ca^2+^/Mg^2+^-free PBS and placed directly in lysis buffer (5mM Tris-HCl, pH 7.0 (LifeTechnologies), 5mM DTT (Thermo Scientific), 0.02% Triton X-100 (Fisher Scientific), 0.5 U/μLRibolock RNAse inhibitor (Thermo Fisher)). Altogether 24 siControl cells from two control embryos and 24 siDUX4 cells from three siDUX4 embryos were collected for library preparation. The library was prepared according to the published protocol (Islam et al., 2012, 2014; Tohonen et al., 2015). The amplified libraries were sequenced on the Illumina HiSeq2500 V2 Rapid mode, using a 60 bp custom read1 primer.

#### Preprocessing and data analysis for STRT RNA-seq

The sequenced STRT raw reads were processed using the STRTprep pipeline (Krjutskov et al., 2016a) (v3dev branch commit 91a62d2 available at https://github.com/shka/STRTprep/tree/v3dev) with Bowtie v 1.1.2.0 (Langmead et al., 2009) and TopHat v.2.1.1 (Kim et al., 2013; Trapnell et al., 2009). Low-quality reads and redundant reads were excluded, and the processed nonredundant reads were aligned to hg19 human reference genome sequences, ERCC spike-in sequences and human ribosomal DNA unit (GenBank: U13369) with RefSeq transcript alignments as a guide of exon junctions. For TFE-based statistics, the mapped reads were assembled according to the alignments, and uniquely mapped reads within the first exons of the assembled transcripts were counted, as described in Tohonen et al., (2015) (Tohonen et al., 2015). For gene-based statistics, uniquely mapped reads within (i) the 5’-UTR or the proximal upstream (up to 500 bp) of the RefSeq protein coding genes, and (ii) within the first 50 bp of spike-in sequences, were counted. Prior to downstream analysis, low quality samples (or cells) were filtered out based on (i) ERCC spike-in read counts, (ii) human genome mapped / ERCC spike-in read counts (this represents the relative abundance of poly (A) transcripts), (iii) percentage of ERCC spike-in 5’-end reads, and (iv) percentage of reads mapping to the 5’-end of coding genes were excluded. Details about these parameters have been provided here:

(https://github.com/shka/STRTprep/blob/master/doc/result.md). Additionally, for the *DUX4* knockdown experiment, the distribution of cells based on the number of coding genes was estimated. Cells below the first quartile Q1 where the number of genes expressed was <1100 were also excluded. After filtering, 12 siControl and 15 siDUX4 cells were used for downstream analysis. Normalization and DE was performed using SAMstrt v 0.99.0 (Katayama et al., 2013; Krjutskov et al., 2016a). Gene counts were normalised to ERCC spike-in counts using the SAMstrt.normalization function with parameter nresamp = 1000. DE between siControl and siDUX4 cells was performed using the SAMseq function with parameters resp.type = 'Two class unpaired', nperms = 1000 and fdr.output = 0.05, thus the significance was calculated using Wilcoxon statistics and the empirical distribution.

#### Gene ontology (GO) analysis

A gene enrichment analysis of GO terms was performed individually for up and downregulated genes using metascape (Zhou et al., 2019) (https://metascape.org/gp/index.html) using default parameters. All genes expressed in siRNA knockdown dataset were used as background. For the ATAC-seq dataset, peaks were annotated to +/− 300bp of the 5’-end of GENCODE v 27 transcripts to identify their corresponding genes. As background, genes which were annotated from all peaks were used.

#### Bioinformatics analysis and molecular dynamics simulations of the DUX4 protein

The sequences of the human DUX family proteins were obtained from the UniProt database (The UniProt Consortium) (UniProt, 2021), whereas DUX4 sequences from other primates were retrieved from the non-redundant database of NCBI(Pruitt et al., 2007) using blastp (Johnson et al., 2008) with human DUX4 (UniProt ID: Q9UBX2) as the query sequence (Key resources table). Multiple sequence alignment over the full-length sequences was carried out using MAFFT (Katoh and Standley, 2013) with default parameters. Secondary structures, solvent accessibility and disordered regions were predicted using SCRATCH (Cheng et al., 2005) and RaptorX-Property (Wang et al., 2016). The 9aaTAD web server (“Most Stringent Pattern” (Piskacek et al., 2007)) was used to predict 9aaTAD motifs. The crystal structure of the DUX4 HD1-linker-HD2 fragment bound to DNA (PDB: 6E8C (Lee et al., 2018)) was obtained from the Protein DataBank (PDB (Berman et al., 2000)). PyMOL (version 2.4; Schrödinger LLC) and Bodil (Lehtonen et al., 2004) were used to analyze inter-HD interactions. For modelling the binding of the 9aaTAD peptide ^371^GLL**L**DE**LL**A^379^ and the KBM ^416^E**Y**RA**LL**^421^ peptide of DUX4 onto the KIX domain, the NMR structure (model 1/20) of human KIX in complex with MLL and pKID peptide (Bruschweiler et al., 2013) (PDB: 2LXT) was chosen as the template; the sequence ^846^PSD**I**MD**FV**L^854^ of MLL and ^13^S**Y**RK**IL**^138^ of pKID were mutated in PyMOL to match the DUX4 sequences ^371^GLL**L**DE**LL**A^379^ and ^416^E**Y**RA**LL**^421^, respectively, and the coordinates of extra residues of the MLL and pKID peptides were removed; PDB coordinates for KIX in complex with DUX4 9aaTAD and KBM peptides in Data S1. MD simulations followed the protocol illustrated elsewhere (Tamirat et al., 2019). Shortly, missing atoms were added with Chimera (Pettersen et al., 2004). The ff14SB (Maier et al., 2015) and OL15 (Zgarbova et al., 2015) force fields of AMBER package (v. 18) (Case et al., 2018) were used for protein and DNA, respectively. Structures were solvated with explicit TIP3P water molecules (Jorgensen et al., 1983). Periodic boundary conditions were applied and the particle-mesh Ewald algorithm was employed for electrostatic interactions (Essmann et al., 1995). Hydrogen bond interactions were monitored using CPPTRAJ (Roe and Cheatham, 2013) and VMD (Humphrey et al., 1996).

#### Expression of hu4man KIX domain from CBP, binding of C-terminal peptides

A synthetic, codon-optimized gene in the pET100/TOPO vector (Invitrogen GeneArt Gene Synthesis, Thermo Scientific) was used to express the human KIX domain of CBP (residues 587-673; Uniprot Q92793) in *E. coli* BL21 DE3 cells. The expressed construct (14.5 kDa) contained 36 extra N-terminal residues, including a 6xHis tag, the XpressTM epitope and an enterokinase cleavage site, in addition to the KIX domain (86 residues). Transformed *E. coli* were grown with ampicillin selection in 600 mL of ZYM-5025 autoinduction medium (Studier, 2005) for 10 h at 37°C. The cells were collected by centrifugation at 3,000×g for 20 min and stored at −20°C. The pellets were thawed and suspended in buffer A (50 mM Tris, pH 8.0, 500 mM NaCl) with 20 mM imidazole and lysed by sonication. The supernatant was separated from the cell debris by centrifugation (45,000×g for 40 min) and applied to a three-step purification protocol using an ÄKTA Pure 25 chromatography system (GE) with a UV detector. First, a Histrap HP (1 mL; GE) column was used for metal-affinity chromatography: the sample was applied to the column and subsequently washed with 25 column volumes (CV) of buffer A with 20 mM imidazole. KIX was eluted with a linear imidazole gradient from 20 mM to 500 mM in buffer A over 15 CV, and the column was then washed with 5 CV of 500 mM imidazole in buffer A. The KIX containing fractions (ca. 7 mL) were identified by UV absorbance at 280 nm, pooled, then dialyzed (30 volumes, two exchanges, CelluSep dialysis membrane, MWCO 6-8K; Membrane Filtration Products, Inc.) against buffer B (25 mM CHES, pH 9.0). Second, anion exchange chromatography was performed with a Resource Q column (1 mL; GE). The cleared (3,200×g for 15 min) dialysis pool was applied to the column, the column was washed with 20 CV of buffer B, and eluted with a linear gradient from 0 to 1 M NaCl in buffer B over 15 CV. The KIX containing fractions were pooled (ca. 4 mL) and concentrated with an Amicon Ultra-4 centrifugal filter (MWCO 3K; Merck Millipore) to a volume of 0.5 mL. Third, the concentrated sample was applied to a Superdex 75 10/300 GL size exclusion chromatography column (GE) and eluted with buffer C (25 mM Tris, pH 8.4, 150 mM NaCl) using a flow rate of 0.5 mL/min (0.5 mL fractions). The purity of the sample was analyzed with SDS-PAGE and Coomassie staining, and the concentration was verified by measuring the UV absorbance at 280 nm with NanoDrop One (Thermo Scientific).

Binding assays were performed using a Monolith NT(TM) microscale thermophoresis instrument (Nanotemper Technologies). The His-tagged KIX domain was labeled non-covalently using the Monolith NT(TM) His-Tag Labeling Kit RED-tris-NTA (1st generation; Nanotemper Technologies) according to manufacturer's instructions. Monolith NT.Automated Capillary Chips (Nanotemper Technologies) were used to test binding and to determine the affinity of the 9aaTAD (^371^GLLLDELLA^379^) and KBM (^416^EYRALL^421^) peptides to KIX; the homeodomain of human LEUTX with His-Tag was used as a negative control. Peptides were ordered from GenScript (key resources table) and dissolved in deionized water. The final concentration of KIX in the assay was 20 nM, and the concentration of each peptide in a binding test assay was 5 μM (250-fold molar excess). The KIX protein and the peptide samples were diluted in PBS-Tween (pH 7.4; 0.05% v/v of Tween 20) buffer for the assays.

#### DUX4 TetOn and DUX4 dCas9 plasmid constructs

Full-length *DUX4* (NM_001293798.2) was synthesized and cloned between the SalI and BamHI sites of the pB-tight-hMAFA-ires-EmGFP-pA-PGK-Puro vector (a kind gift from Dr. Diego Balboa, Stem Cells and Metabolism Research Program, University of Helsinki) at GenScript (Genscript, NJ, USA). For the dCas9-DUX4-C construct, the C-terminal part of the DUX4 was PCR amplified from the pB-tight-hDUX4-ires-EmGFP-pA-PGK-Puro vector (using primer sequences as listed in the key resource table) and cloned into the CAG-dCas9 VP192-GIP plasmid in place of the VP192 domain.

#### Cloning of DUX4 and DUX4 KBM mutant to MAC-tag gateway destination vector

DUX4 was first amplified in a two-step PCR reaction from pB-tight-DUX4-ires-EmGFP-pA-PGK-Puro and cloned into a Gateway compatible entry clone using Gateway BP Clonase II (Invitrogen) according to manufacturer’s instructions. The entry clone was further cloned to Gateway compatible destination vectors containing the C-terminal MAC-tag (Addgene #108077) as described (Liu et al., 2018). Transfection and selection of the Flp-In™ T-REx™ 293 cells (Invitrogen, Life Technologies, R78007, cultured in manufacturer’s recommended conditions) and affinity purification of the final product was done as previously (Liu et al., 2018). DUX4 KBM mutant was amplified in a PCR reaction from the pB-tight-DUX4-ires-EmGFP-pA-PGK-Puro plasmid using primers that eliminate the DUX4 C-terminal KBM motif (key resources table). The following steps of the Gateway cloning was performed as in case of the wildtype DUX4. As a negative control a GFP sequence with nuclear localization signal was cloned in the MAC-tag gateway vector instead of DUX4 wildtype or DUX4 KBM mutant.

#### Liquid chromatography-mass spectrometry

Analysis was performed on a Q-Exactive mass spectrometer with an EASY-nLC 1000 system via an electrospray ionization sprayer (Thermo Fisher Scientific), using Xcalibur version 3.0.63. Peptides were eluted from the sample with a C18 precolumn (Acclaim PepMap 100, 75 μm × 2 cm, 3 μm, 100 Å; Thermo Scientific) and analytical column (Acclaim PepMap RSLC, 65 μm × 15 cm, 2 μm, 100 Å; Thermo Scientific), using a 60 min buffer gradient ranging from 5% to 35% Buffer B, then a 5 min gradient from 35% to 80% Buffer B and 10 min gradient from 80% to 100% Buffer B (0.1% formic acid in 98% acetonitrile and 2% HPLC grade water). 4 μL of peptide sample was loaded by a cooled autosampler. Data-dependent FTMS acquisition was in positive ion mode for 80 min. A full scan (200–2000 m/z) was performed with a resolution of 70,000 followed by top10 CID-MS^2^ ion trap scans with a resolution of 17,500. Dynamic exclusion was set for 30 s. Database search was performed with Proteome Discoverer 1.4 (Thermo Scientific) using the SEQUEST search engine on the Reviewed human proteome in UniProtKB/SwissProt databases (http://www.uniprot.org, downloaded Nov. 2018). Trypsin was selected as the cleavage enzyme and maximum of 2 missed cleavages were permitted, precursor mass tolerance at ±15 ppm and fragment mass tolerance at 0.05 Da. Carbamidomethylation of cysteine was defined as a static modification. Oxidation of methionine, and in BioID samples biotinylation of lysine and N-termini were set as variable modifications. All reported data were based on high-confidence peptides assigned in Proteome Discoverer (FDR <0.05).

#### Data analysis of affinity purification data

Significance Analysis of INTeractome (SAINT (Choi et al., 2011))-express version 3.6.3 (Teo et al., 2014)and Contaminant Repository for Affinity Purification (CRAPome, http://www.crapome.org) were used to discover statistically significant interactions from the AP-MS data (Mellacheruvu et al., 2013). The DUX4 LC-MS data (from one experiment, 4 replicates) was analyzed using SAINTexpress alongside a large dataset of other transcription factors, as well as a large GFP control set. Significance threshold for a statistically significant interaction was set as BFDR score lower than 0.05.

Overrepresentation analysis of statistically significant interactions matching protein complex database CORUM (Giurgiu et al., 2019) (https://mips.helmholtz-muenchen.de/corum/) and Gene Ontology terms was performed using R-package enrichR (Chen et al., 2013). Protein interaction networks were constructed from statistical significant (BFDR <0.05) protein-protein interactions imported to Cytoscape 3.6.0(Shannon et al., 2003). Known prey-prey interactions were obtained from the iRef database (http://irefindex.org). The negative control (GFP) samples were treated similarly as the DUX4 samples (tetracycline induction in the case of AP-MS and biotin treatment in the case of BioID-MS). The data from the negative control MS runs are summarized in Table S4.

### Quantification and statistical analysis

No statistical methods were applied to pre-determine sample sizes. Statistical analysis was performed using R version 3.6.1 or Microsoft Excel (*t*-test). The statistical test and the number of replicates for each analysis is described in the figure legends or STAR methods section. A p-value < 0.05 was considered significant.

## Data Availability

Accession numbers for data generated in this paper and weblinks to the code have been listed in the key resources table. The ATAC-seq, CAGE/NET-CAGE, and bulk STRT data have been deposited in Gene Expression Omnibus (GEO: GSE171803). Cloned transcript sequences have been deposited in European Nucleotide Archive (ENA: LR694082-LR694089). Any additional information required to reanalyze the data reported in this paper is available from the lead contact upon request.

## Supplemental information

Document S1. Figure S1–S9

Table S1. A supplementary table showing the NET-CAGE data read summary, raw counts, log2 counts per million, and differential expression of promoters and enhancers, related to Figures 1, S3, and S5

Table S2. A supplementary table showing the summary of the bulk STRT dataset Read-level statistics, raw and log10 normalised gene counts with differential expression values are shown. Related to Figure 1 and S4.

Table S3. A supplementary table showing the summary of the DUX4 knockdown STRT dataset Read-level statistics, raw and log10 normalised gene counts, and significantly differentially expressed genes are shown. Related to Figures 3 and S6.

Table S4. A supplementary table showing transient (BioID) and stable (AP-MS) DUX4 protein-protein interactions and negative control (GFP) counts, related to Figures 4, S7 and S8

Data S1. A supplementary data showing coordinates (KIX-9aaTAD-KBM.pdb) for modelled KIX in complex with DUX4 9aaTAD and KBM peptides, related to Figures 4 and S9

Data S2. A supplementary data showing coordinates (DUX4_HD1-HD2.pdb) of DUX4 HD1-HD2 without bound DNA at the end of a 100 ns molecular dynamics simulation, related to Figures 4 and S9
