# Supplementary material for: DUX4 is a multifunctional factor priming human embryonic genome activation
Source: iScience. 2022 Mar 22;25(4):104137. doi: 10.1016/j.isci.2022.104137 (PMC8990217; doi:10.1016/j.isci.2022.104137)
Supplement: Document S1. Figure S1–S9 [file mmc1.pdf]

iScience, Volume *25*

Supplemental information

DUX4 is a multifunctional factor priming human embryonic genome activation

Sanna Vuoristo, Shruti Bhagat, Christel Hydén-Granskog, Masahito Yoshihara, Lisa Gawriyski, Eeva-Mari Jouhilahti, Vipin Ranga, Mahlet Tamirat, Mikko Huhtala, Ida Kirjanov, Sonja Nykänen, Kaarel Krjutškov, Anastassius Damdimopoulos, Jere Weltner, Kosuke Hashimoto, Gaëlle Recher, Sini Ezer, Priit Paluoja, Pauliina Paloviita, Yujiro Takegami, Ai Kanemaru, Karolina Lundin, Tomi T. Airenne, Timo Otonkoski, Juha S. Tapanainen, Hideya Kawaji, Yasuhiro Murakawa, Thomas R. Bürglin, Markku Varjosalo, Mark S. Johnson, Timo Tuuri, Shintaro Katayama, and Juha Kere

Figure S1. Characterization of *DUX4* TetOn hESC clones. Related to Figure 1.

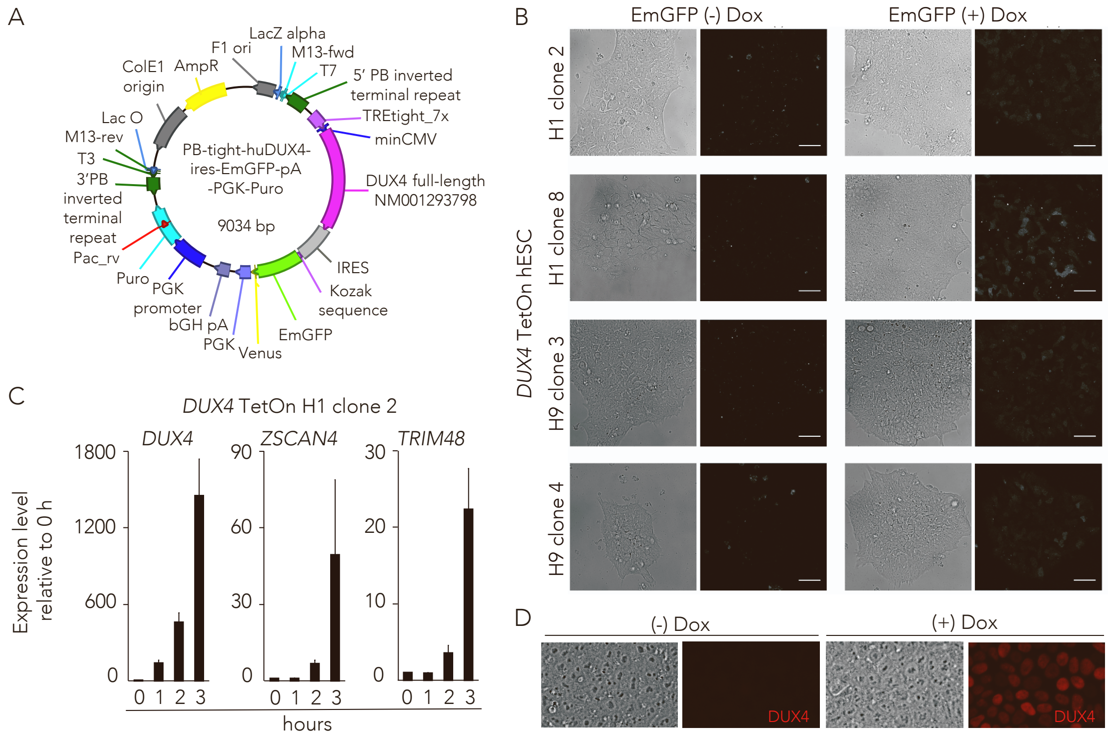

(A) A map of *DUX4*-ires-EmGFP piggyBac vector.

(B) *DUX4* TetOn hESC H1(WA01) clones 2 and 8, and H9 (WA09) clones 3 and 4, live-imaged after +/- 3-hour doxycycline treatment. EmGFP (indicating DUX4 expression) shown in grey. Scale bars 20 µm.

(C) mRNA expression level kinetics of *DUX4*, *ZSCAN4*, and *TRIM48* relative to 0-hour after 1-hour, 2-hour, and 3-hour doxycycline induction measured using qPCR. Data are shown as mean ±SEM. Expression levels are shown for the *DUX4* TetOn H1 clone 2, from three independent cell cultures +/- doxycycline treatment as indicated. Similar expression patterns were found for other DUX4 TetOn hESC clones.

(D) *DUX4* TetOn hESCs +/- 4-hour doxycycline-treatment immunostained for *DUX4* (MAb E5-5). Representative images for nuclear DUX4 staining are shown for the H1 clone 2. Similar staining patterns were seen for the H1 (WA01) clone 8 and H9 (WA09) clones 3 and 4.

Figure S2. ATAC–gained peaks are enriched for developmental processes. Related to Figure 1.

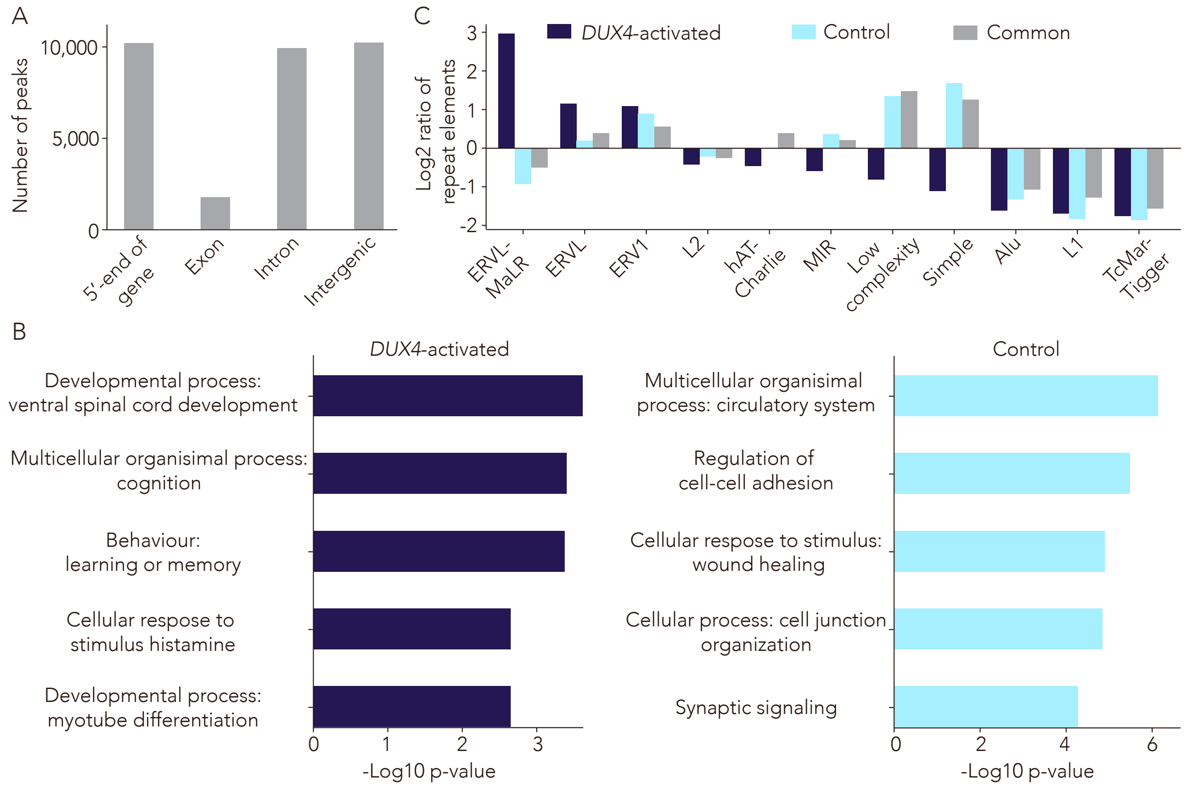

(A) Bar plot showing the distribution of ATAC–seq common peaks across the genome.
(B) Gene ontology term enrichment analysis depicting the five terms with the most significant p-values for biological processes in *DUX4*-activated (left) and control (right) peaks. *DUX4*-activated peaks are enriched for developmental processes such as myotube differentiation.
(C) Bar plot showing the log2 ratio of ATAC–seq peaks overlapping repeat elements over randomly selected background regions. *DUX4*-activated peaks are specifically enriched for ERVL–MaLR and depleted for most other repeat elements.

Figure S3. *DUX4*–activated promoters and enhancer-like regions are specifically enriched for the ERVL-MaLR element and not for most other repeat elements. Related to Figure 1.

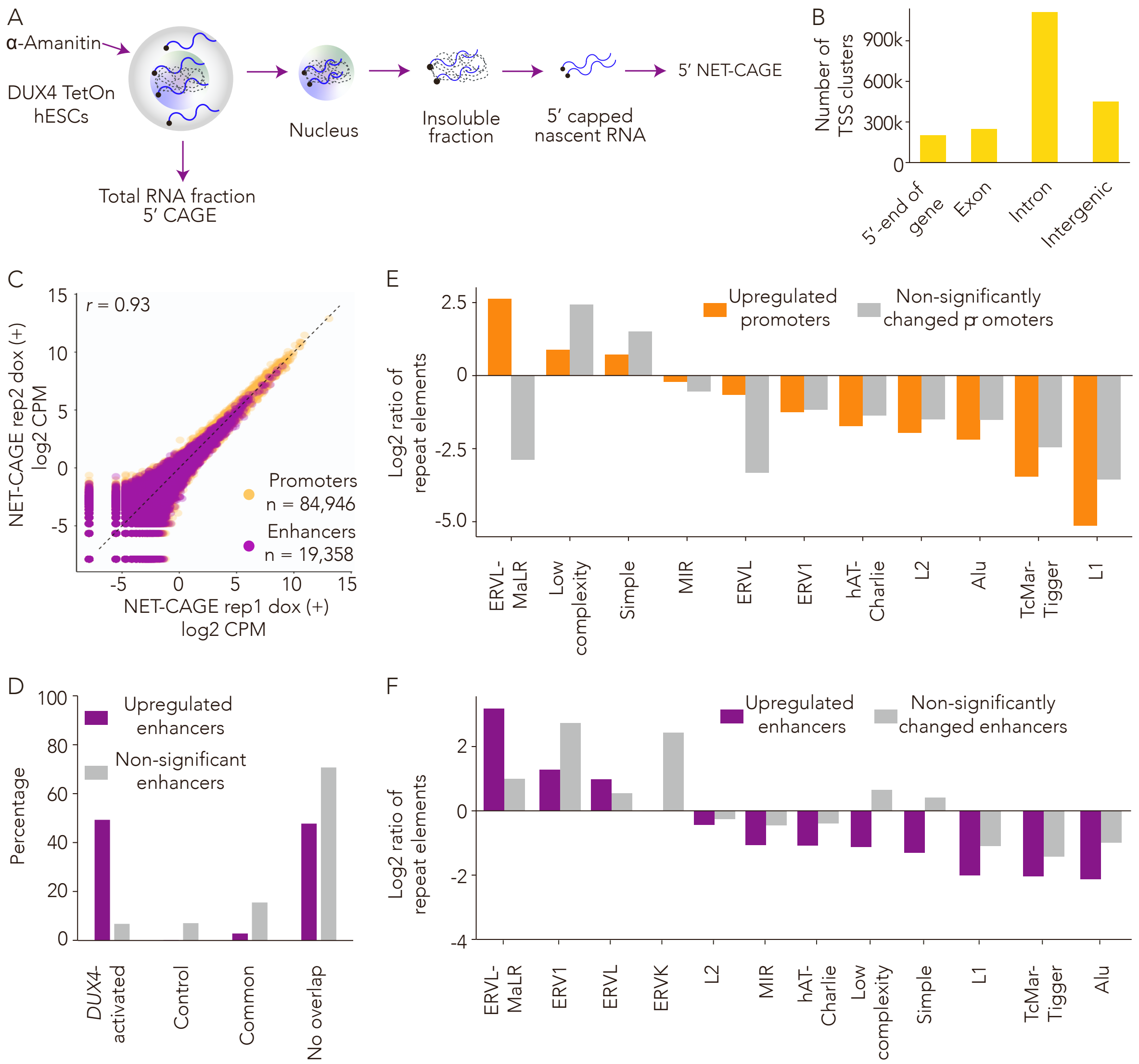

(A) Schematic illustrating principle of the Native Elongating Transcript – Cap Analysis of Gene Expression (NET-CAGE) methodology. Nascent RNA is purified via subcellular fractionation and is sequenced from the 5′ –end.

(B) Bar plot showing the number of transcription start site (TSS) clusters used to identify promoters and putative enhancer-like regions.

(C) Reproducibility between two NET–CAGE dox (+) biological replicates.

Spearman correlation was 0.93. Orange dots, promoters; purple dots, enhancers.

(D) Bar plot showing the percentage of putative enhancer-like regions overlapping ATAC-seq peaks.

(E and F) Bar plot showing the log2 ratio of promoters (D) and enhancers (E) overlapping repeat elements over randomly selected background regions.

Figure S4. Putative *DUX4* target genes cloned from human 4-cell stage embryos. Related to Figure 1.

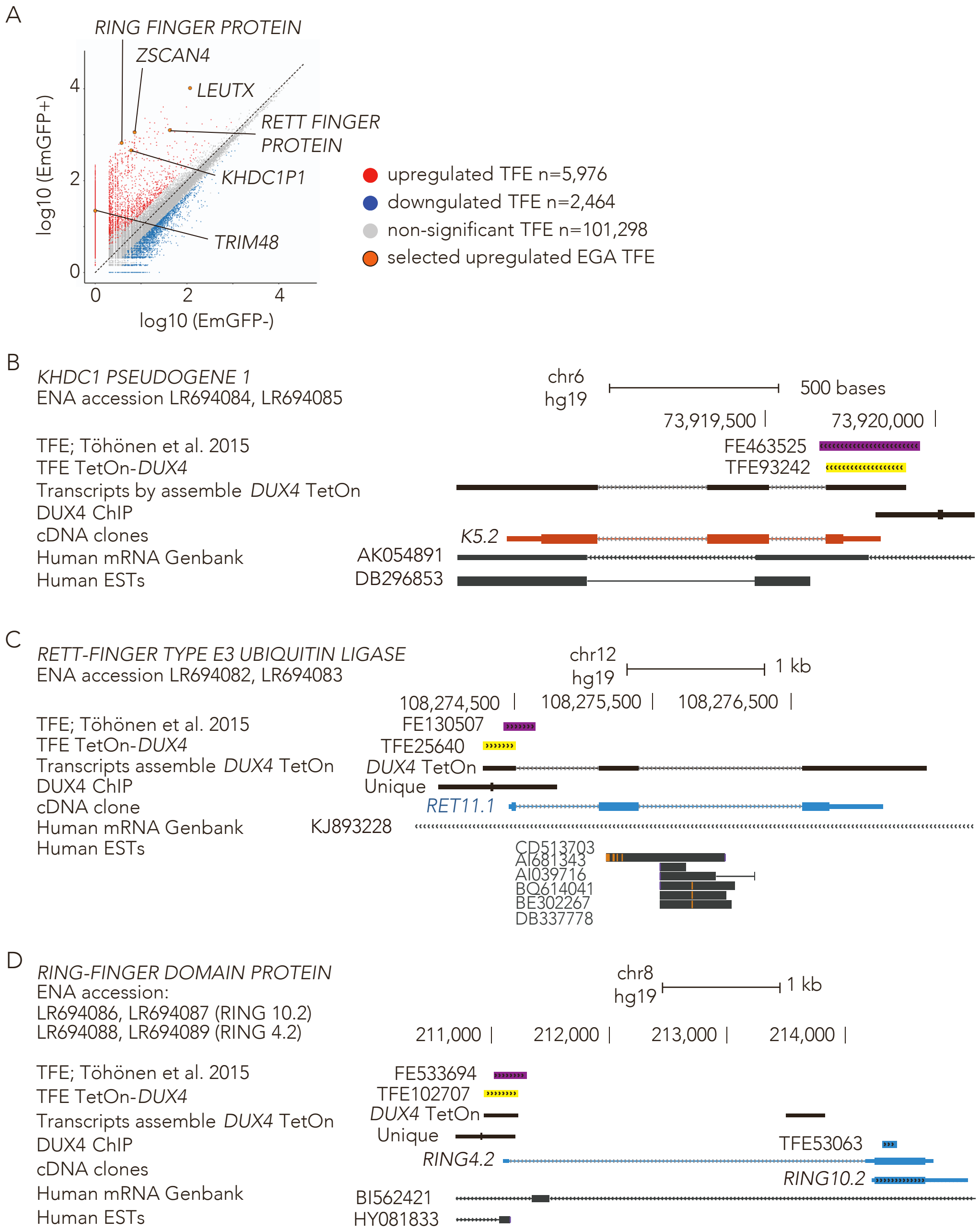

(A) A scatter plot showing the comparison between EmGFP (-) and *DUX4*-activated EmGFP (+) cells using bulk STRT RNA-seq. Red and blue dots indicate significantly upregulated and downregulated TFEs, respectively. Grey dots indicate non-significantly differentially expressed TFEs. Significantly differentially expressed TFEs are defined using the following thresholds: q-value < 0.05, fluctuation < 0.05 and differential expression score ≥ 2 or ≤ 0.5. Significance was calculated using the Wilcoxon test. Data is shown for *DUX4* TetOn H1 clone 2 (n=3 +/- EmGFP) and 8 (n=3 +EmGFP, n=2 -EmGFP), and DUX4 TetOn H9 clones 3 (n=3 +EmGFP, n=2 -EmGFP) and 4 (n=3 +EmGFP, n=2 -EmGFP) that were FACS-selected (+/- EmGFP) and collected per indicated condition.

(B) Predicted *KHDC1 PSEUDOGENE 1* (clone K5.2), at chromosome 6 (73,918,461-824 73,920,115) was expressed by human 4-cell stage embryos (FE463525) (Tohonen et al., 2015)

and upregulated in *DUX4* expressing hESCs (TFE93242). TFEs overlapped with DUX4 binding sites (DUX4 ChIP; GSE33838) (Geng et al., 2012). The cDNA clone K5.2 (thick orange labelling indicates exons and grey thin labelling indicates introns) was cloned from the 4-cell stage embryo. The clone corresponds to the *KHDC1 PSEUDOGENE 1* transcript assembly in *DUX4* expressing cells.

(C) Putative *RETT-FINGER TYPE E3 UBIQUITIN LIGASE* at chromosome 12 (108,273,771-831 108,277,850) was expressed by human 4-cell stage embryos (FE130507) (Tohonen et al., 2015) and upregulated in *DUX4* expressing hESCs (TFE25640). The DUX4 ChIP-seq peak (Geng et al., 2012) overlapped with the TFEs. RET11.1 was cloned from human 4-cell stage embryo (clone RET11.1). Thick blue labelling indicates exons and thin grey labelling indicates introns.

(D) Putative *RING-FINGER DOMAIN PROTEIN* at chromosome 8 (210,701-215,100) was expressed by human 4-cell stage embryos (TFE533694) (Tohonen et al., 2015) and upregulated in DUX4 expressing hESCs (TFE102707). DUX4 ChIP-seq; GSE33838 (Geng et al., 2012) overlapped with the TFEs. Two cDNA clones, RING 4.2 and RING 10.22, were cloned from the human 4-cell stage embryo. Thick blue labelling indicates exons and grey thin labelling indicates introns. For all putative *DUX4* targets, transcript assemblies (mRNA Genbank and human ESTs), including un-spliced isoforms, are shown. Abbreviations: TFE, transcript 5' far end; EST, expressed sequence tag.

Figure S5. Validation of the putative enhancer for *KHDC1P1*. Related to Figure 1.

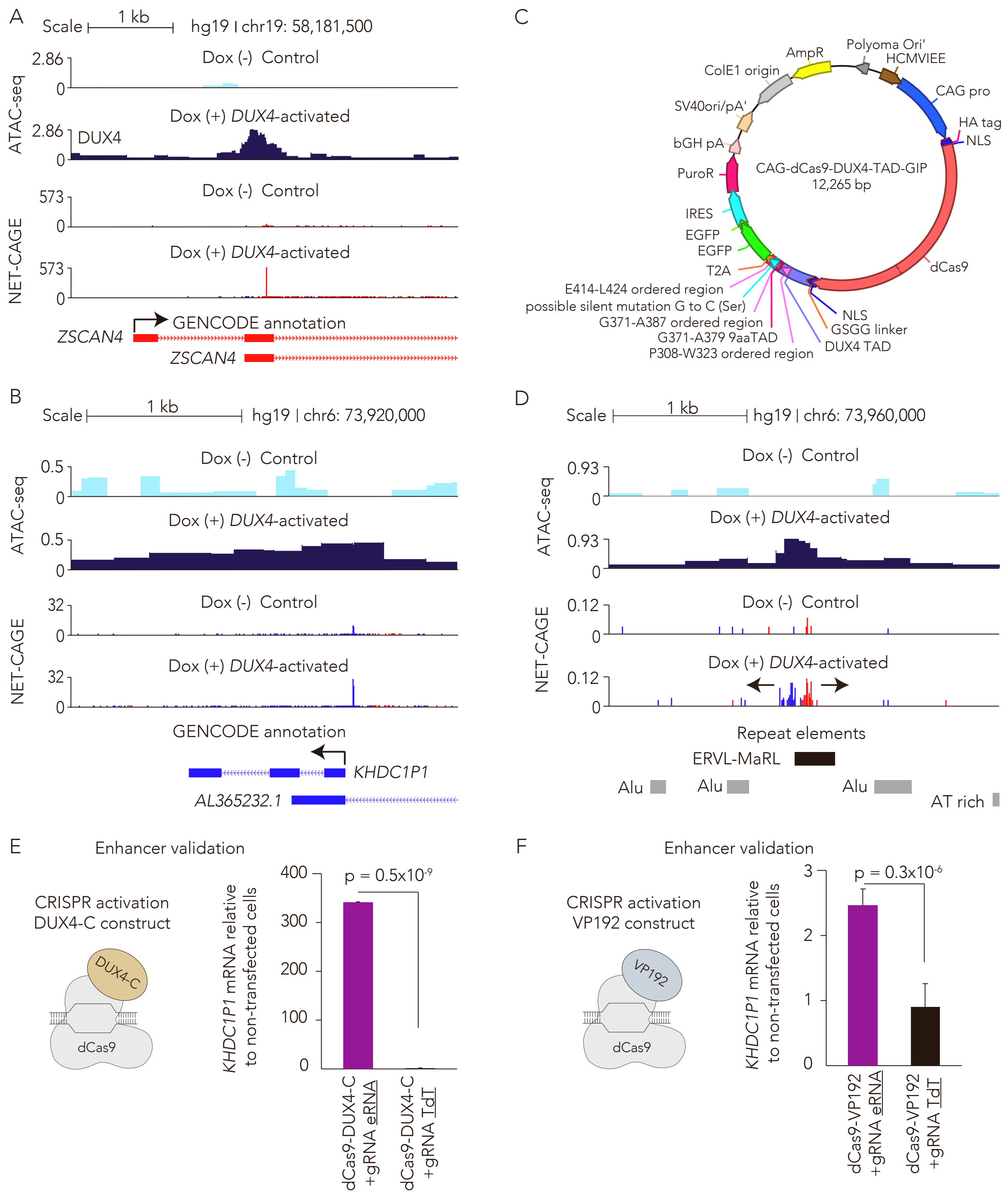

(A) Genome browser views showing the promoter for *ZSCAN4* with significantly higher accessibility (ATAC–seq) and expression (NET–CAGE) in dox (+) samples. NET–CAGE reads in red, plus strand; NET–CAGE reads in blue, minus strand.
(B) Similar to (A) but showing the promoter of the *KDHC1P1* gene.
(C) A map of dCas9-DUX4-C fusion vector.
(D) Genome browser view showing the putative enhancer of *KHDC1P1*. NET-CAGE signal shows bidirectional transcription start sites of enhancer RNAs in dox (+) samples. The putative enhancer also overlaps an ERVL-MaLR repeat element.
(E and F) Schematic of CRISPR dCas9 activator constructs fused with the DUX4 C-terminal end (E) or VP192 (F) and used in combination with guide RNA pools to activate putative enhancers. Graphs show the *KHDC1P1* expression level relative to non-transfected cells (n=6 from independent cell cultures (E); n=6 from independent cell cultures (F)). Guide RNA construct for TdT were used as the negative control (n=6 from independent cell cultures). Data are shown as mean ± SD and p-values were calculated using two-tailed Student's *t*-test.

Figure S6. Integration with publicly available RNA-seq datasets in human, mouse and macaque. Related to Figure 2 and Figure 3.

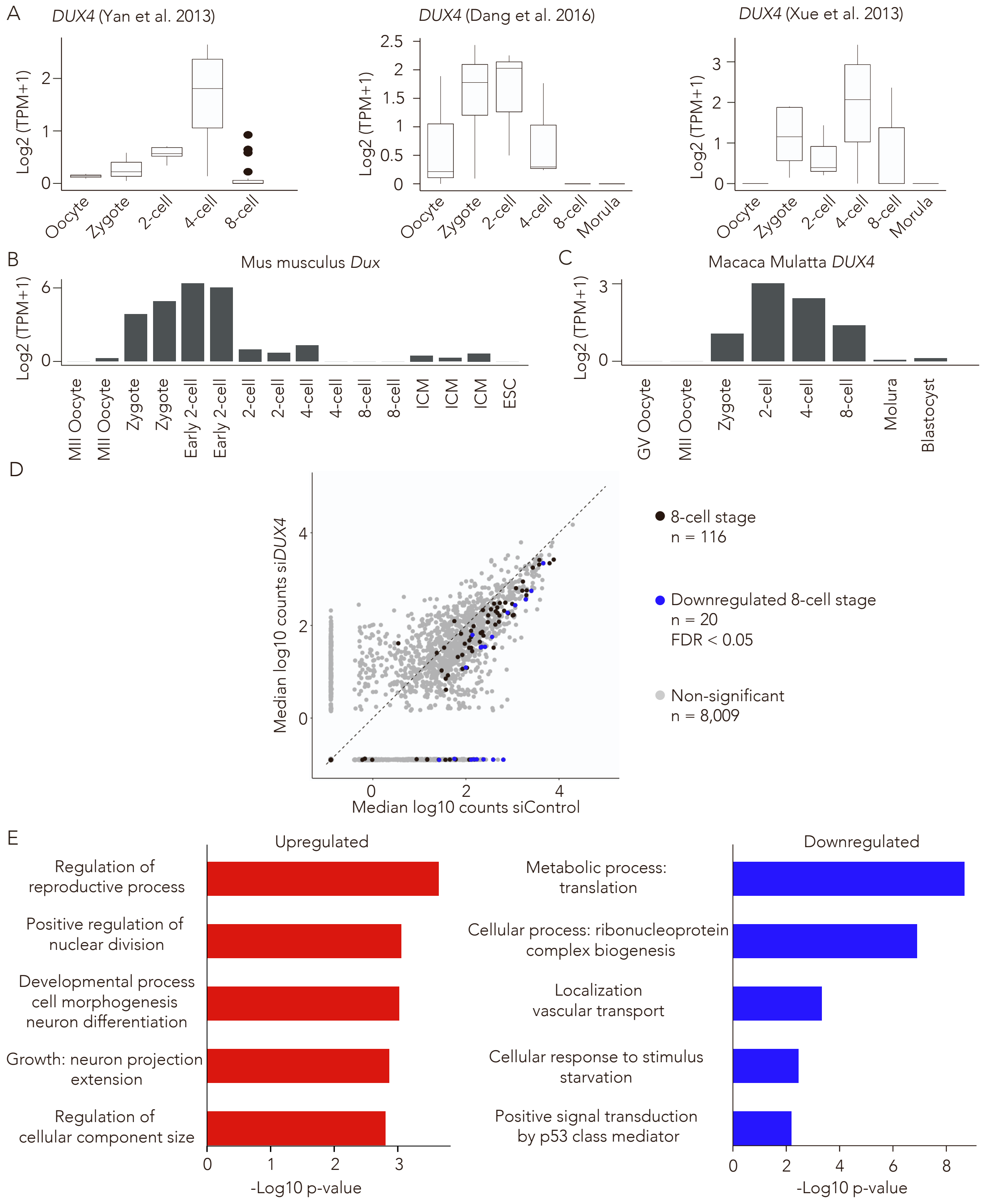

(A) Box plots showing *DUX4* mRNA expression in human oocytes, zygotes and cleavge stage embryos. Source data: (Yan et al., 2013) (GSE36552), (Xue et al., 2013) (GSE44183) and (Dang et al., 2016) (GSE71318). In each box, the median is indicated, the edges are the 25th and 75th percentiles, the whiskers extend to the data points not considered outliers.

(B and C) Bar plots showing expression of mouse *Dux* (B) and Macaca Mulatta *DUX4* (C) oocytes, zygotes and cleavage stage embryos.

(D) Scatter plot showing the comparison of median log10 gene expression levels in the siControl (n = 12) versus siDUX4 (n = 15) blastomeres. Black dots represent known 8-cell stage genes (Tohonen et al., 2015). Blue dots represent significantly downregulated 8-cell stage genes in the siDUX4 blastomeres compared to the siControl blastomeres. Significance was calculated using Wilcoxon test, FDR < 0.05. Grey dots represent non-significantly differentially expressed genes.

(E) Gene ontology term enrichment analysis depicting the five terms with the most significant p-values for biological processes in genes upregulated (left) and downregulated (right) following siRNA knockdown of *DUX4* in human embryos.

Figure S7. DUX4 protein-protein interactions identified using the MAC-tag method. Related to Figure 4.

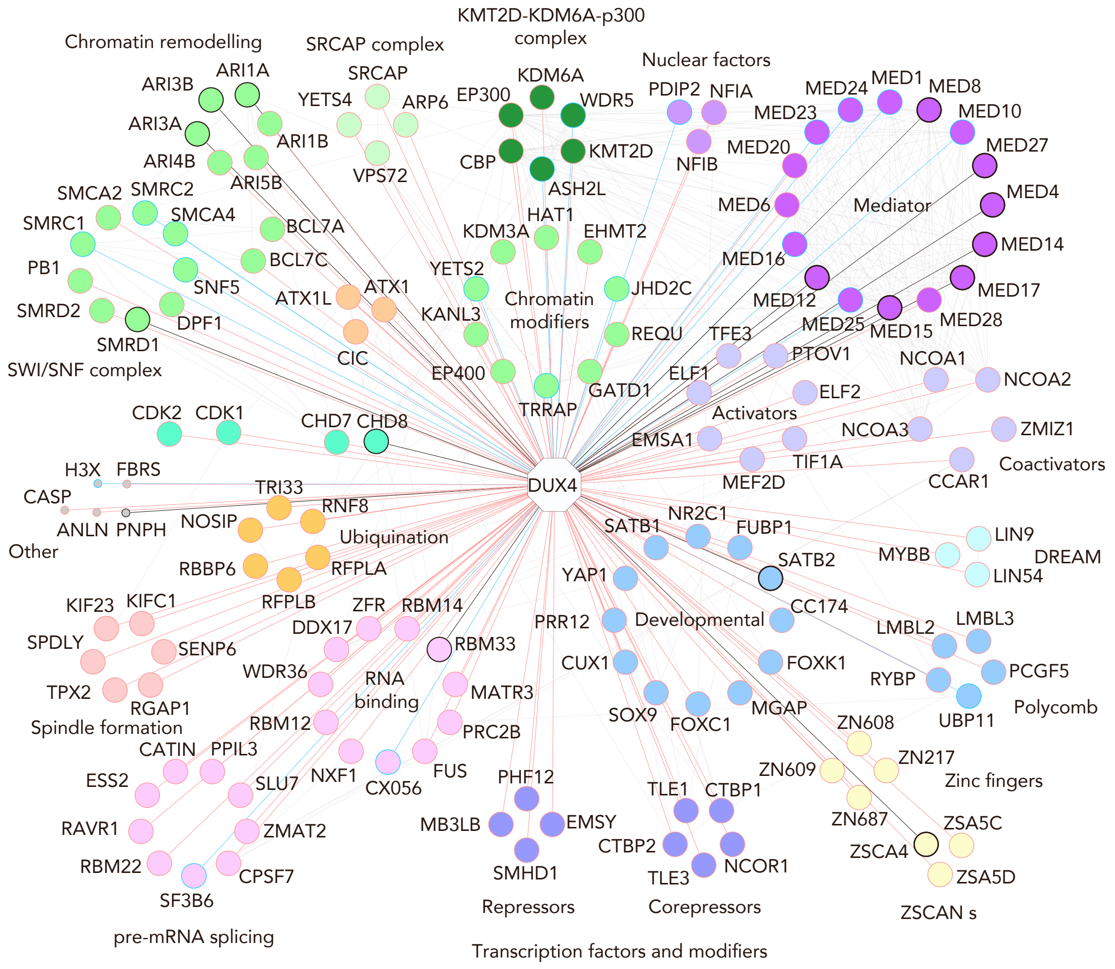

Transient BioID-MS interactions are indicated with red lines and stable AP-MS -interactions are indicated with blue lines. Proteins that appeared in both data sets are indicated with a black line and outline. Known prey-prey interactions shown in grey (iREF).

Figure S8. Expression levels of DUX4 protein interactions in human oocytes and embryos. Related to Figure 4.

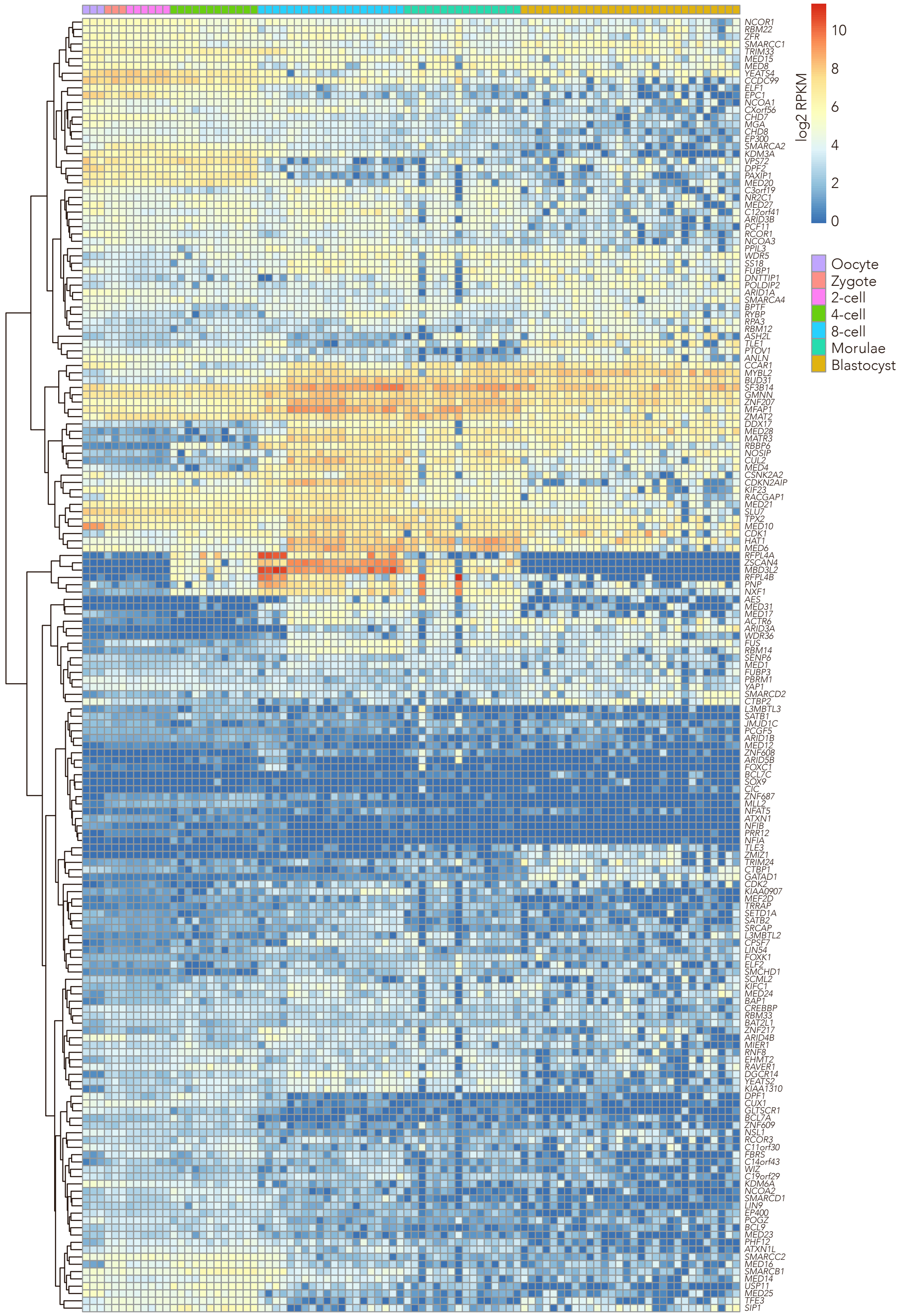

A heatmap showing RNA expression levels (Yan et al., 2013) (GSE36552) of the DUX4 protein-protein interactors.

Figure S9. Microscale thermophoresis binding analysis of peptides to human KIX domain and interactions of DUX4. Related to Figure 4.

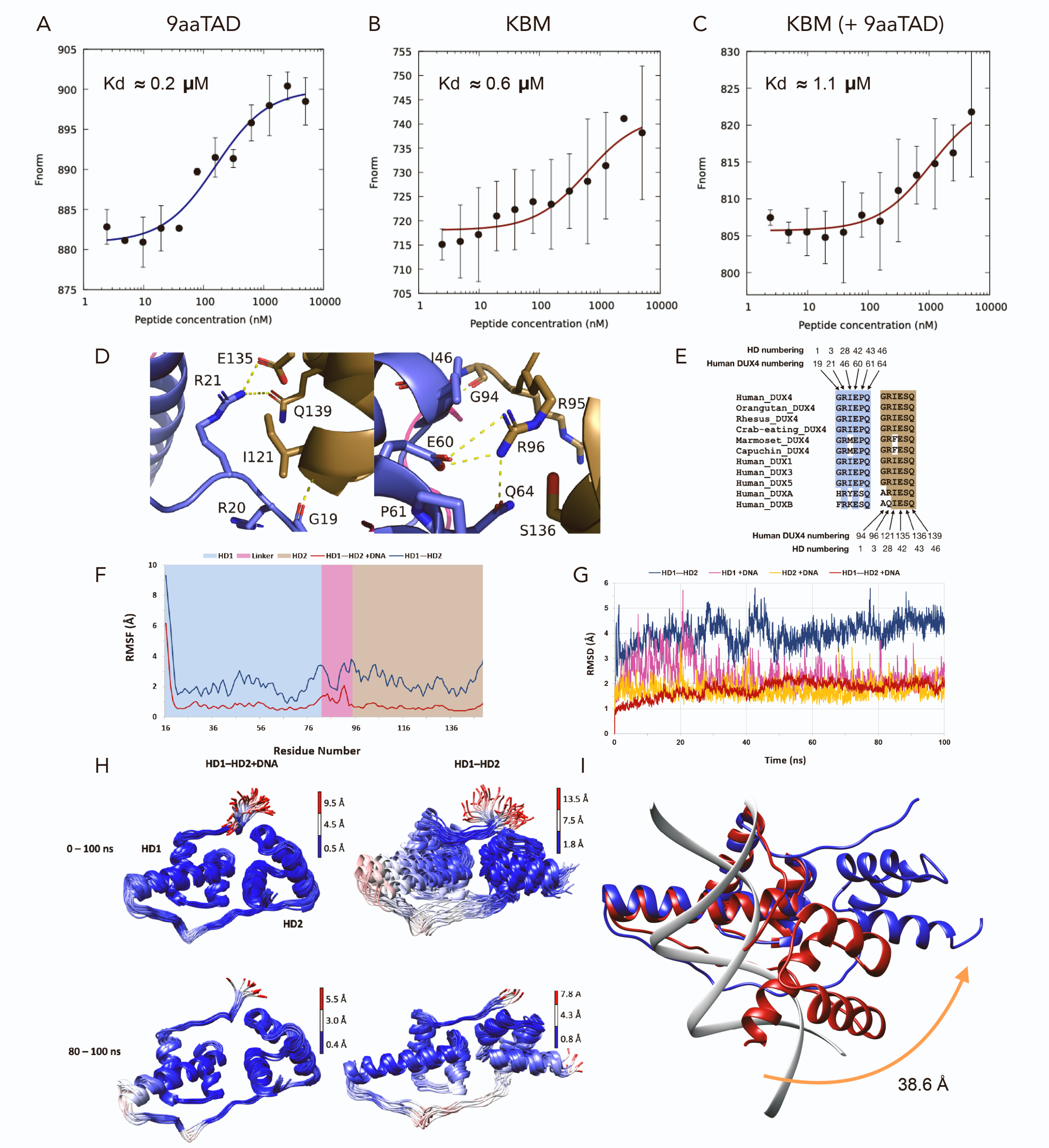

(A–C) 9aaTAD peptide (C370-Q386) (A), KBM peptide (E414-E423) (B), and KBM peptide with saturating 9aaTAD peptide to KIX (C).

(D) Inter-HD interactions stabilizing DUX4 HD1 and HD2 in the absence of bound DNA.

(E) Sequence comparison of HD1-HD2 interacting residues seen in human DUX4 with other primates and other human double HD transcription factors.

(F) RMSF (Cα atoms) of X-ray structure of DUX4 with (red curve) and without (blue curve) bound DNA during a 100 ns MD simulation. HD1 (blue), linker (magenta) and HD2 (gold).

(G) RMSD (backbone atoms) with reference to starting conformation of X-ray structure of DUX4 HD1-HD2 with and without bound DNA, and separately for HD1 and for HD2 with

bound DNA, during 100 ns MD simulations.
(H) Superposed conformations of DUX4 with (left) and without (right) DNA, sampled during 100 ns (top) and final 20 ns (bottom) of the simulation. Chain traces are colored based on the Cα-atom RMSD relative to the median structure at 50 ns or 90 ns. DNA-bound DUX4 shows higher stability than DNA-free DUX4; both exhibit larger fluctuations at the unconstrained N-termini and linker loops. A more stable conformation of DNA-free DUX4 exposing residues of the recognition helices was attained during the last 20 ns.
(I) Final pose, DNA-free DUX4 (blue), after 100 ns simulation with HD1 superposed on HD1 of DNA-bound DUX4 X-ray structure (red and grey), revealing the degree of "opening" seen in the simulation; e.g. the Cα-atom of R146 of the third helix of HD2 differs in relative position by 38.6 Å. Abbreviations: TAD, transactivation domain; HD, homeodomain; KBM, KIX-binding motif.
